# “Spice”-related deaths in and around Munich, Germany: A retrospective look at the role of synthetic cannabinoid receptor agonists in our post-mortem cases over a seven-year period (2014–2020)

**DOI:** 10.1007/s00414-023-02995-2

**Published:** 2023-04-19

**Authors:** Olwen Groth, Gabriele Roider, Verena Angerer, Jan Schäper, Matthias Graw, Frank Musshoff, Volker Auwärter

**Affiliations:** 1 Institute of Forensic Medicine, University of Munich, 80336 Munich, Germany; 2 Institute of Forensic Medicine, Medical Center, University of Freiburg, 79104 Freiburg, Germany; 3 Institute of Forensic Medicine, Forensic Toxicology, St. Gallen Cantonal Hospital, 9010 St. Gallen, Switzerland; 4 Forensic Science Institute, Bavarian State Criminal Police Office (BLKA), 80636 Munich, Germany; 5 Forensic Toxicological Center (FTC) Munich, Dessauerstrasse 13-15, 80992 Munich, Germany; 6 Faculty of Medicine, University of Freiburg, 79110 Freiburg, Germany

**Keywords:** Synthetic cannabinoid receptor agonists (SCRAs,“Spice”), Post-mortem toxicology, Fatal intoxication, Toxicological Significance Score (TSS), New psychoactive substances (NPS)

## Abstract

Synthetic cannabinoid receptor agonists (SCRAs, “Spice”) are a diverse group of recreational drugs, with their structural and pharmacological variability still evolving. Forensic toxicologists often rely on previous reports to assess their role in intoxication cases. This work provides detailed information on the “Spice”-related fatalities around Munich, Germany, from 2014 to 2020. All cases underwent an autopsy. Pharmaceutical and illicit drugs were detected and quantified in post-mortem peripheral blood or liver by liquid chromatography-tandem mass spectrometry (LC–MS/MS). Based on circumstantial evidence, only those cases for which a prior consumption was suspected underwent additional analyses for SCRAs and other new psychoactive substances in post-mortem blood, liver or antemortem specimens. Drug concentrations, pathological findings at autopsy and case histories were considered to assess and rank the SCRAs’ involvement in each death. Concentration ranges for the individual substances in blood were defined and their distribution patterns over the investigated period were determined and correlated with their legal status and local police seizures. We identified 41 different SCRAs among 98 fatalities. 91.8% were male, at a median age of 36 years. SCRAs played a causative role in 51%, contributory role in 26%, and an insignificant role in 23% of cases. In correlation with local police seizures and legal status, 5F-ADB was the most prevalent in our cases, followed by 5F-MDMB-PICA and AB-CHMINACA. Cumyl-CBMICA and 5F-MDMB-P7AICA were among the least frequently detected SCRAs. “Spice”-related fatalities and SCRAs’ causative role have significantly decreased among our cases since the German New Psychoactive Substances Act.

**Supplementary Information:**

The online version contains supplementary material available at 10.1007/s00414-023-02995-2.

## Introduction

JWH-018 (1-pentyl-3-(1-naphthoyl)indole) [1] was one of the first synthetic cannabinoids, commonly referred to as “Spice,” to be identified in “illegal” preparations destined for recreational use [2, 3]. Following this discovery, several countries added the compound to their list of restricted substances in an attempt to hamper further distribution for its use as a psychoactive drug [4, 5]. However, these restrictions only marked the beginning of a seemingly never-ending cat and mouse game between legal authorities and the producers of such drugs [6, 7].

JWH-018 was soon exchanged for structural analogues on the recreational drug market, which, too, were replaced by newer ones not long after. Slight adaptations to the chemical structure made it relatively easy for drug producers to circumvent a recently implemented restriction. Consequently, the German drug market alone saw the addition of at least eight other structurally similar substances merely three years after the first synthetic cannabinoids had been identified [8, 9]. In return, Germany and other countries implemented generic legislations, restricting whole groups of substances with structural or pharmacological similarities instead of adding individual substances to an already lengthy list of banned drugs [10, 11]. Finally, a significant decrease in the annual number of newly emerging drugs could be observed. However, the change in the legal control of synthetic cannabinoids also led drug producers to take an even more creative approach to further introduce “non-illegal” substances to the market. Thus, also the structural diversity of synthetic cannabinoids is evolving [12], and the addition of new compounds continues [13], albeit in lower numbers than in earlier years. As a result, by the end of 2020, a total of 209 synthetic cannabinoid drugs have been reported to the European Monitoring Centre for Drugs and Drug Addiction (EMCDDA) [5].

One of the major problems associated with such a large and diverse subgroup of psychotropic drugs is the wide range of activity profiles between the individual substances. Like the phytocannabinoid Δ^9^-tetrahydrocannabinol (THC), synthetic cannabinoids act as agonists on the endogenous cannabinoid receptor system. However, the synthetic counterparts, many of which exhibit full agonistic activity and stronger affinities for the CB_1_ and CB_2_ receptors, typically show a much higher potency. Especially during the initial frequent emergence of new compounds, even minor changes in the chemical structure could significantly alter the extent of the pharmacological effect, making the potential side-effect profiles of newer substances highly unpredictable.

The international drug-abusing population has experienced synthetic cannabinoids with toxicities ranging from the relatively “insignificant” harmful effects of Cumyl-PEGACLONE [14] to the numerous fatal outcomes associated with 5F-ADB consumption [15, 16]. Furthermore, the cardiotoxic effects exhibited by some SCRAs are seemingly more pronounced than for others [17, 18]. Generally, cardiovascular effects include arrhythmia, myocardial infarction and hypertension [19]. Other common adverse events observed for most synthetic cannabinoids include respiratory depression, sedation, nausea and vomiting, as well as seizures and psychosis. These effects either directly or indirectly pose the risk of serious life-threatening conditions with potentially fatal outcomes [20].

For the forensic toxicologist, information about the deceased’s medical history, former drug abuse habits, the circumstances of death and the presence of other drugs in their system are some of the essential requirements to determine the role of a synthetic cannabinoid in a “Spice”-related death [21]. Furthermore, the knowledge of blood or tissue concentrations of the drug at the time of death are required to correlate it with the extent of the anticipated toxic effects. Unlike therapeutically approved drugs, novel recreational drugs typically have very limited available pharmacological data that indicate potentially toxic or fatal concentrations. Thus, forensic toxicologists must rely on previous reports of intoxication cases to assess the role of “Spice” in a death case. However, information on synthetic cannabinoid concentrations in post-mortem blood and their corresponding toxic effects is rather limited. Considering that “Spice” is still widely available over the internet at low costs, these drugs continue to pose serious health threats and still play a relevant role in fatal intoxications.

All these factors warrant better availability of information about their toxicological significance in former cases to help forensic toxicologists interpret results from synthetic cannabinoid-related deaths successfully. We thus created an overview of all autopsy cases at our institute from seven consecutive years, for which at least one synthetic cannabinoid could be detected post-mortem. Our main aim was to assess the toxicological significance [22] of the detected synthetic cannabinoids in the respective fatalities. Furthermore, we evaluated the chronological change in the appearance and the gradual decrease or increase in the number of the respective substances in these cases over the investigated period for its correlation with their local market availability.

## Materials and methods

### Study design

All cases included in this retrospective study underwent a post-mortem investigation at the Institute of Forensic Medicine in Munich between 2014 and 2020. All autopsy cases at the Munich Institute of Forensic Medicine for which a potential role of prescription and/or illicit drugs are suspected in the fatality undergo routine toxicological analyses to detect pharmaceutical substances and classical drugs of abuse, including alcohol. Based on circumstantial data and case histories, additional targeted analyses for the detection of synthetic cannabinoids and other new psychoactive substances (NPS) are performed only on those cases for which a prior consumption is indicated or suspected. Of these, all autopsy cases for which one or more synthetic cannabinoid receptor agonists could be identified during the investigated period were evaluated for the purpose of this study.

#### Toxicological significance score

Case files from the corresponding public prosecutor’s office were examined for demographic and other relevant information about the deceased. Together with the autopsy findings, toxicological results and information from published case reports, these data were applied to each case to assess the toxicological significance of the detected synthetic cannabinoids according to the method proposed by Elliott et al. [22]. Drugs other than the synthetic cannabinoid under review were also considered when performing this assessment. Due to the limited availability of comparative concentration datasets, synthetic cannabinoid blood concentrations were compared between cases from our institute to estimate potentially toxic and lethal levels of that drug. Where available, data from literature regarding concentration ranges were also applied. As is often true for post-mortem forensic casework, a reasonable assessment of the deceased’s tolerance to the substance is generally only possible through hair analyses. Such analyses usually do not form part of routine casework, unless specifically indicated.

In accordance with the method published by Elliott et al. [22], a low toxicological significance score (TSS 1) was assigned to cases for which low concentrations of the drug and an unrelated cause of death could be identified. Cases, both with and without an alternative cause of death, for which comparatively moderate concentrations were detected, but the substance may still have contributed to the fatality, obtained a higher score (TSS 2). A typical example is a fatal intoxication with high concentrations of other central nervous system depressants combined with relatively moderate concentrations of the synthetic cannabinoid. In contrast, a TSS of 3 was assigned to those cases where the synthetic cannabinoid was most likely the leading cause of death. These also include cases where the death resulted from adverse behaviour (e.g., jumping from a high building), which may have been subsequent to a synthetic cannabinoid triggered psychosis or anxiety. Cases for which the role of the synthetic cannabinoid was uncertain were generally assigned the higher of the two scores. For example, if it were uncertain whether a TSS of 1 or a TSS of 2 should be assigned to a case, TSS 2 was used.

This evidence-based assessment was performed independently by two forensic toxicologists. Differences in the assigned scores were discussed in detail until an agreement could be reached.

#### Evaluating the course of the appearance and fading of synthetic cannabinoids in post-mortem cases over the investigated period

The total number of synthetic cannabinoids detected and how these individual substances were scattered over the investigated period (i.e. how often a particular substance was detected each year) were determined. The latter was correlated with the change in legal status and the number of police seizures in the German state Bavaria that were analysed at the Bavarian State Criminal Police Office (BLKA). The concentration range for each synthetic cannabinoid detected in femoral venous blood was defined. For cases where cardiac blood was used due to the unavailability of femoral venous blood (e.g. due to post-mortem decomposition), the concentrations detected in cardiac blood were compared to femoral blood concentrations of the relevant substances.

#### Case details

Demographic data, including age and gender, of all cases for which at least one synthetic cannabinoid could be detected, were evaluated. A detailed summary of all positive cases, including quantitative toxicological data, case histories, the most relevant findings at autopsy, the leading cause of death and the TSS assigned to each case, was created. The time span between death and sampling of biological specimens was determined based on the time of death provided by medical officials and the date and time of the autopsy. For cases with an unknown time of death, an estimate of the post-mortem interval was made based on the interval between the time the person was last seen alive and discovery of the body, as well as the condition of the corpse at autopsy.

### Toxicological analysis

#### Sample collection

Biological specimens for toxicological analysis were either collected at autopsy or taken from hospital samples where relevant and available. Post-mortem femoral venous blood was used to detect and quantify pharmaceutical substances and illicit drugs, including synthetic cannabinoids, by liquid chromatography-tandem mass spectrometry (LC–MS/MS). For cases where no femoral venous blood was available or where otherwise indicated (e.g., due to hospitalization prior to death), other blood samples (cardiac blood, antemortem whole blood or serum), liver, muscle tissue or urine were used. Liver and muscle tissue (2.0 g) were homogenized with 10 mL of isotonic sodium chloride (NaCl) solution for 5 min in an ULTRA-TURRAX^®^ (IKA, Staufen, Germany) before sample extraction.

#### Liquid chromatography-tandem mass spectrometry for detecting synthetic cannabinoids

The analysis for the detection of synthetic cannabinoid agonists was performed at the Institute of Forensic Medicine Freiburg. Sample preparation was performed using a two-step liquid–liquid extraction procedure. Following the addition of internal standard solution, carbonate buffer (pH 10) and extraction mixture I (hexane:ethyl acetate, 99:1, *v/v*) to blood, liver homogenate or urine, the sample was gently mixed and centrifuged. The supernatant was transferred to an HPLC vial. Extraction mixture II (hexane:ethyl acetate, 80:20, *v/v*) was added to the residue, and mixing and centrifugation were repeated. The supernatant (1 mL) was added to the first extract. The combined extracts were evaporated to dryness, reconstituted in 100 µL of acetonitrile containing ammonium formate solution (1% acetonitrile, 0.1% formic acid and 2 mM ammonium formate in water:acetonitrile, 0.1% formic acid and 2 mM ammonium formate, 80:20, *v/v*) and analyzed by LC–MS/MS.

LC–MS/MS analysis was performed according to a previously published method [23]. The mass spectrometric identification of all analytes was based on at least two transitions per analyte. The method was updated regularly, using information obtained from an ongoing systematic online monitoring procedure of the drug market [13].

It should, however, be considered that the applied method was validated for serum. This poses a potential limitation for the analysis of post-mortem blood or liver, seeing that differences in matrix effects between these matrices and serum may lead to elevated measurement uncertainty [16]. Furthermore, the chemical instability of some synthetic cannabinoid receptor agonists, particularly those comprising labile ester functions (e.g., 5F-ADB and 5F-MDMB-PICA), may hinder their detection.

#### Liquid chromatography-tandem mass spectrometry for detecting pharmaceutical substances and illicit drugs (not including synthetic cannabinoids)

Sample preparation and analysis for detecting pharmaceutical substances and illicit drugs, excluding synthetic cannabinoids, were performed at the Forensic Toxicological Center (FTC) Munich. Standard procedures were applied according to previously published methods, including the addition of a mixture of internal standards before sample extraction [24, 25].

Blood and homogenized liver were subjected to a protein precipitation step before analysis. For protein precipitation, 100 µL of blood or liver homogenate were treated with 1 mL of acetonitrile, vortexed for 1 min, and centrifuged for 5 min. The supernatant was evaporated to dryness under nitrogen at 37 °C, reconstituted in 150 µL of methanolic ammonium formate solution (5 mM ammonium formate in water and methanol, 85:15, *v/v*), and analyzed by LC–MS/MS. Instrumental details are summarized elsewhere [25].

#### Analysis for ethanol

The analysis for ethanol and methanol was performed at the Institute of Forensic Medicine Munich. Ethanol was quantified in urine and femoral venous blood, or muscle if the latter was unavailable. The average alcohol concentration was determined by using two separate methods, i.e., headspace gas chromatography with flame ionization detector (GC-FID) (Clarus 500, PerkinElmer, Rodgau, Germany) and an alcohol dehydrogenase (ADH) method.

## Results

**Fig. 1:**
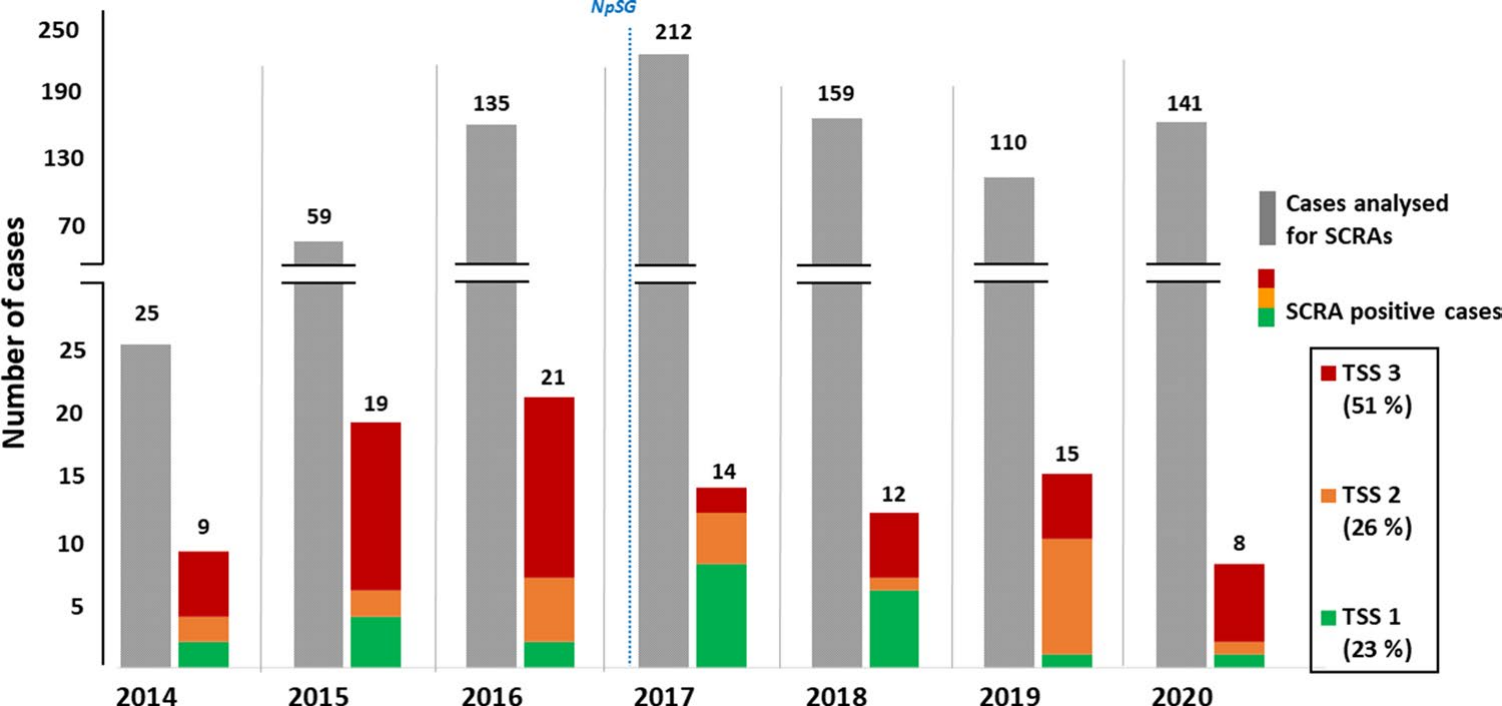
Cases investigated for synthetic cannabinoids from 2014 to 2020. All positive cases are divided according to their toxicological significance scores (TSS). (*NpSG*: New Psychoactive Substances Act in Germany)

A total of 15,240 autopsies (between 2078 and 2293 cases each year) were performed at the Institute of Forensic Medicine Munich from 2014 to 2020. Approximately 25% of these cases (total: 3,931; between 493 and 621 per year) underwent general toxicological analyses for the detection of pharmaceutical substances and illicit drugs, including classical drugs of abuse and alcohol. Of these, additional analyses for the detection of synthetic cannabinoids were performed on 841 cases. A summary of how these cases are distributed over the seven investigated years, including the number of positive cases and the distribution of their toxicological significance scores, is shown in Fig. 1.

**Fig. 2:**
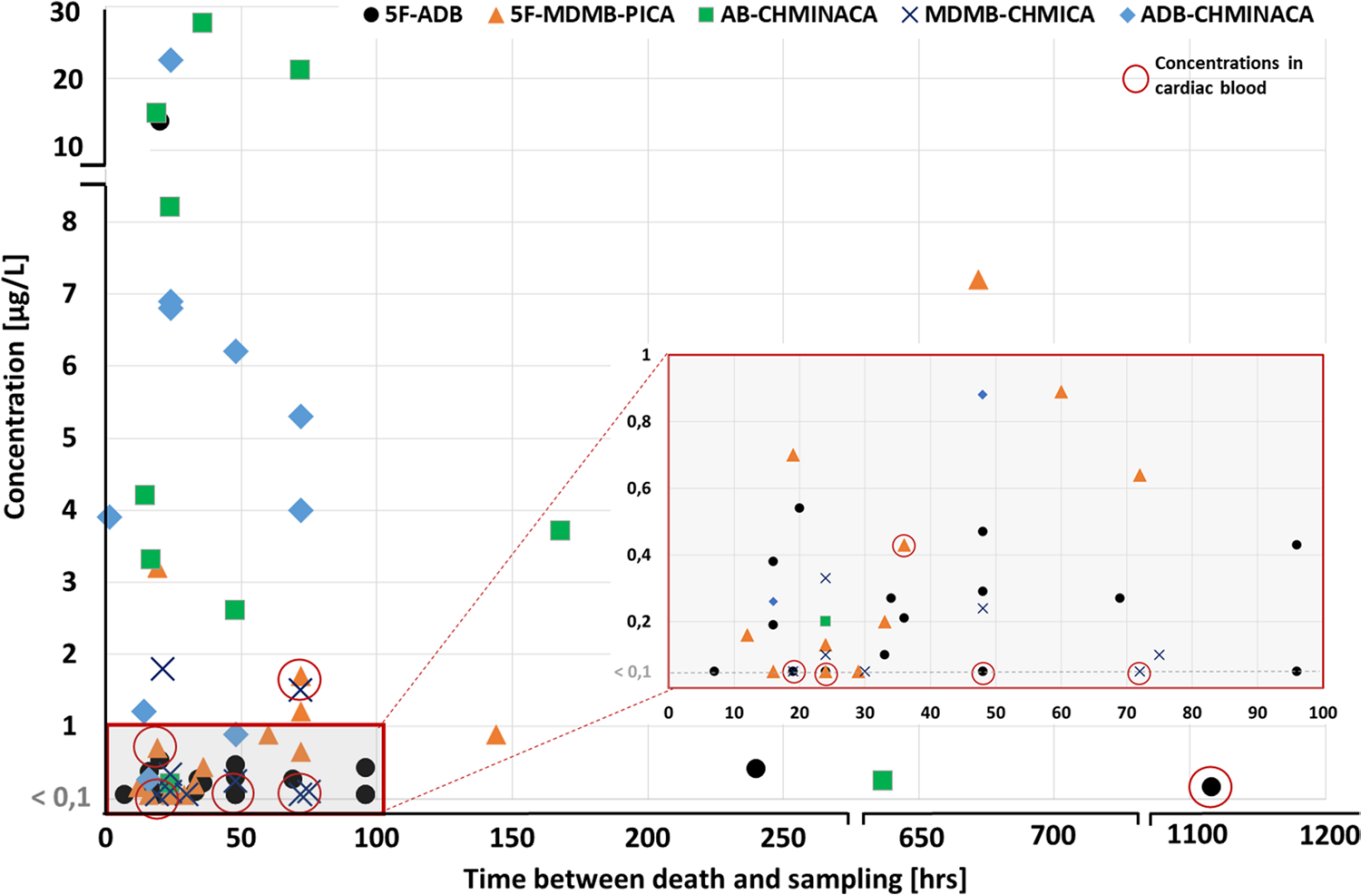
Concentrations of the five most frequently detected synthetic cannabinoids in femoral venous blood or cardiac blood (encircled) plotted against the estimated time elapsed between death and sample collection

Altogether 98 post-mortem cases (11.6% of all cases analysed for SCRAs) could be identified for which at least one synthetic cannabinoid could be detected, 81 in which the synthetic cannabinoids were found in post-mortem femoral venous blood. In cases where femoral venous blood was not available as post-mortem specimen, synthetic cannabinoids were detected in cardiac blood (12 cases) or liver (2 cases). The remaining three persons died in hospital, for whom antemortem blood (2 cases: serum and whole blood respectively) and urine (1 case), that were taken during hospitalization, were analyzed. A detailed summary of all 98 cases can be found in the supplementary information and includes the age and sex of the deceased, the case history, the leading cause of death and the most relevant findings at autopsy (see Tables 1–4 in the supplementary information section). Quantitative data for the synthetic cannabinoid(s) and other relevant drugs, as well as alcohol concentrations in blood or muscle, and urine are also included. The estimated time delay between death or the intoxication incident and sample collection ranged from 1.5 h to approximately 47 days. Figure 2 shows the concentrations of the five most frequently detected synthetic cannabinoids as a function of the estimated time delay between death or the intoxication incident and sampling.

Most of the deceased persons were male (90 cases, 91.8%), with only eight females present in this population (8.2%). The ages ranged from 16 to 63 years for males and 21 to 48 years for females. Both groups had an average age of 36 years and a median age of 35 years.

The most common cause of death was polydrug intoxication with synthetic cannabinoids and other central nervous system depressants combined. A summary of the causes of death for all cases is given in Table 1 below. During autopsy, significant macroscopic pathological changes to the heart, including pronounced cardiac hypertrophy, could be found in nearly 20% of cases (19 cases), irrespective of the cause of death. The youngest person affected in this regard was 16 years old. The deceased was known to have consumed large amounts of synthetic cannabinoids chronically.

**Table 1:** Main causes of death for all cases in which synthetic cannabinoids were detected

Leading cause of death Total cases: 98	Number of cases	%
Polydrug intoxications that include synthetic cannabinoids	38	38.8%
Intoxications with mainly synthetic cannabinoids and alcohol combined	16	16.3%
Intoxications with drugs other than synthetic cannabinoids	16	16.3%
Intoxications with synthetic cannabinoids alone	14	14.3%
Aspiration after synthetic cannabinoid consumption, some also incl. other drugs	9	9.2%
Polytrauma after intoxication (2 after jumping or falling from a high building, 1 traffic accident)	3	3.1%
Drowning, likely due to a synthetic cannabinoid intoxication	1	1.0%
Unknown cause of death	1	1.0%

**Fig. 3:**
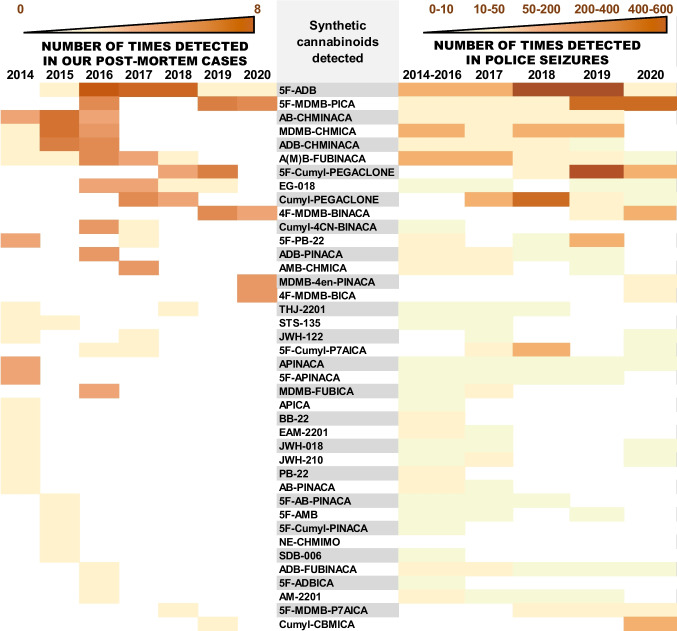
Synthetic cannabinoids detected in post-mortem cases from the Institute of Forensic Medicine Munich and their prevalence between 2014 and 2020 (left) compared to the number of police seizures analysed at the Bavarian State Criminal Police Office (right)

A total of 41 synthetic cannabinoids could be detected among the 98 positive cases. The distribution of these substances over the investigated period is summarized in Fig. 3 (left). The number of police seizures of these substances in the German state of Bavaria that were analysed at the Bavarian State Criminal Police Office (BLKA) is also listed in Fig. 3 (right). 5F-MDMB-PICA and AB-CHMINACA were the two most common substances involved in fatal intoxications with synthetic cannabinoids alone. 5F-ADB was the most common synthetic cannabinoid in deaths due to a mixed intoxication with synthetic cannabinoids and alcohol combined.

**Fig. 4:**
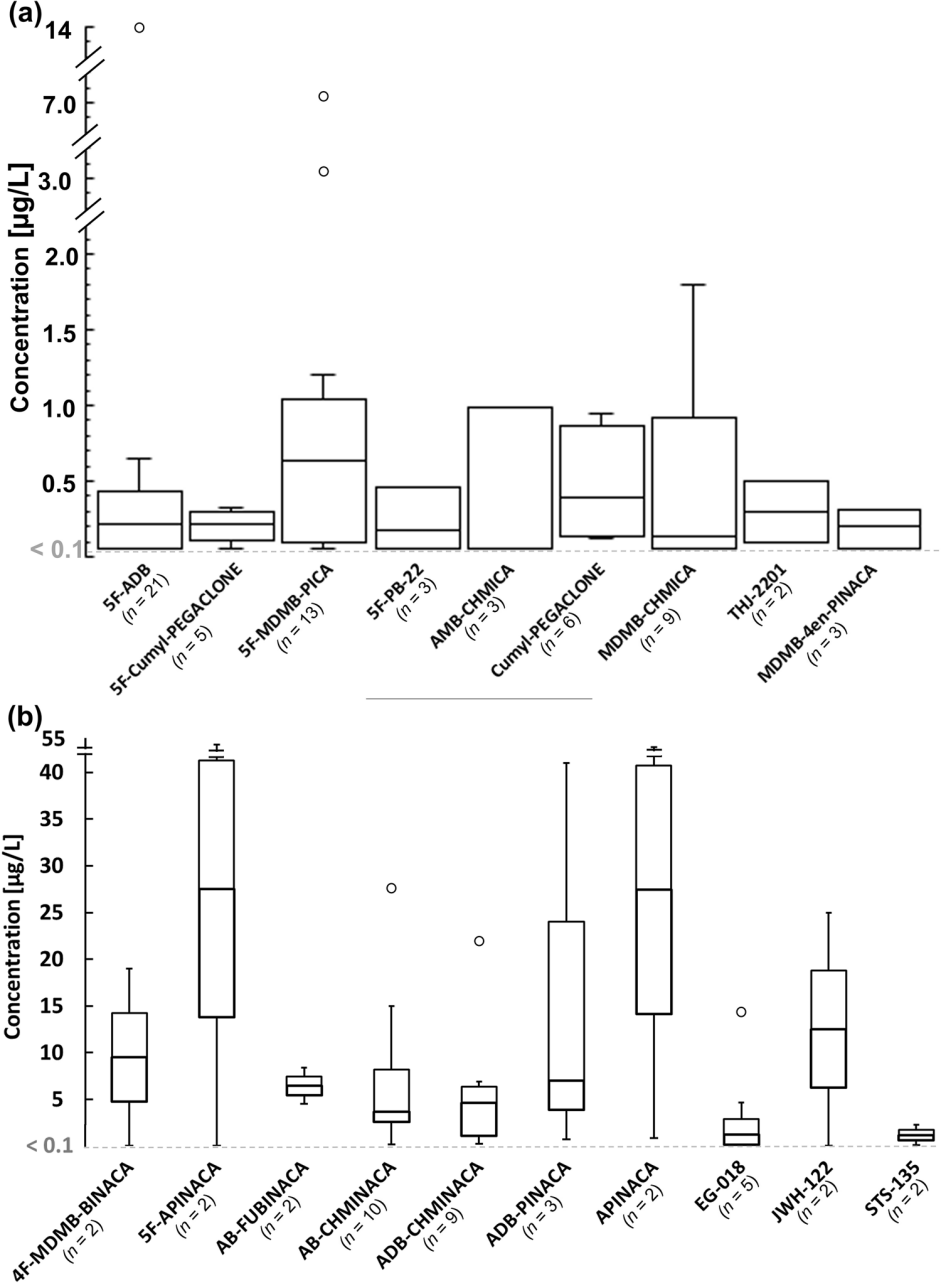
Concentrations of synthetic cannabinoids detected in the (**a**) lower and (**b**) upper concentration ranges (mean ± SD; µg/L) in femoral venous blood

The concentration ranges for all synthetic cannabinoids that were detected in at least two cases in femoral venous blood can be seen in Fig. 4.

## Discussion

Our awareness of the importance of synthetic cannabinoids in fatal intoxication cases has clearly increased since 2014, as is evident from our numbers of toxicological analyses for their post-mortem detection. However, despite more analyses being performed, a clear drop in positive cases could be observed after 2016 (see Fig. 1). This is most likely a reflection of changes in local and international control measures [26], including the New Psychoactive Substances Act (*Neue-psychoaktive-Stoffe-Gesetz,* NpSG), which was enacted in Germany in November 2016. This new generic control of NPS was implemented to compensate for some shortcomings of the German Narcotics Law (*Betäubungsmittelgesetz, BtMG*) and thus to counteract the repetitive appearance of new, uncontrolled substances. However, these generic control measures focused on indole (e.g., JWH-018), indazole (e.g., ADB-FUBINACA) and benzimidazole (e.g., FUBIMINA) based synthetic cannabinoid structures in the beginning, thus not including substances with a tricyclic core scaffold, such as the γ-carbolinones or carbazoles [27, 28]. This legal loophole led to the arrival of yet another generation of synthetic cannabinoid receptor agonists on the recreational drug market merely two weeks after the NpSG came into effect.

Cumyl-PEGACLONE (SGT-151), 5F-Cumyl-PEGACLONE, and Cumyl-CBMICA are typical examples of synthetic cannabinoid drugs that were specifically designed to bypass the law, of which Cumyl-PEGACLONE was the first γ-carbolinone structure to enter the “legal high” market [13]. In our cases, we first observed it in 2017, with a total of six appearances until the first quarter of 2018. Five of these cases were assigned a TSS of 1, underlining Cumyl-PEGACLONE’s relatively insignificant role in intoxication cases, as was also found in an earlier study [14]. In June 2018, it was replaced in our cases by its structural analogue 5F-Cumyl-PEGACLONE, reaching a maximum the following year (see Fig. 3). This, too, probably resulted from the change in legislation, considering that Cumyl-PEGACLONE was added to the annex of the German Narcotics Law in July 2018 [29, 30]. Compared to Cumyl-PEGACLONE, most of our cases with 5F-Cumyl-PEGACLONE were assigned a higher toxicological significance score (TSS 2). This finding is not only in accordance with the theory that fluorine-substituted cannabinoid derivatives generally exert a higher potency [31], but also agrees with earlier case studies regarding the toxic effects of 5F-Cumyl-PEGACLONE [32]. Cumyl-CBMICA was first detected towards the end of the investigated period. The carbazole-based synthetic cannabinoid EG-018 appeared among our cases in October 2016, thus several months before the γ-carbolinones reached the market. EG-018 played a causative role in only one of the six fatalities for which it could be detected. In literature, EG-018 is described as a low-efficacy cannabinoid receptor agonist [33].

Judging from the toxicological significance scores assigned for synthetic cannabinoids from our 98 positive cases, SCRAs have been causative in the death (TSS 3) of more than half of this cohort, most of which occurred before 2017. It played at least a contributory role (TSS 2) in over one-quarter of cases. The most common cause of death was polydrug intoxication with synthetic cannabinoids and other central nervous system depressant drugs combined, several involving heroin. This is not surprising, as multiple-drug consumption is common on the substance-abuse scene [34, 35]. Among the 98 cases, one cause of death could not be determined. It is possible that the deceased, who had a history of drug abuse, had consumed (new) psychoactive substances that were either fatal in very low concentrations, or not at all covered by our analyses. Several cases included fatal intoxications involving only alcohol and synthetic cannabinoids, particularly so for the indazole-based substance 5F-ADB (5F-MDMB-PINACA).

The prevalence of 5F-ADB-related fatalities by far exceeded those deaths with other synthetic cannabinoids amongst our cases, followed by its indole counterpart 5F-MDMB-PICA (see Fig. 3). This higher number of cases with 5F-ADB and 5F-MDMB-PICA may first of all reflect their potencies [15], especially considering that most of these cases received a TSS of 3. 5F-ADB has been described as one of the most dangerous synthetic cannabinoids on the market [36]. It was also one of the most frequently detected synthetic cannabinoids in a review of eight case series and 26 case reports on “Spice”-related fatalities by Giorgetti et al. [16]*.* Likewise, 5F-MDMB-PICA is also described as a highly potent cannabinoid receptor agonist in literature, which is associated with several cases of serious adverse health effects and deaths [37, 38]. The detection of the less potent substances like Cumyl-PEGACLONE in our post-mortem cases was thus mostly incidental, considering that an alternative cause of death could almost always be identified.

In addition to their potency, the detection frequency of individual synthetic cannabinoid agonists over the investigated period also correlates with their market availability in the Munich area. As was observed for our post-mortem cases, 5F-ADB and 5F-MDMB-PICA were also the two substances most frequently detected in seized preparations. In contrast to most synthetic cannabinoids, the change in legal status of these two substances had little effect on their market availability. Nearly three years after its inclusion in the German Narcotics Law, 5F-ADB was still widely distributed in our area. It was not until 5F-ADB was placed under international control towards the end of 2018 that its availability dropped. However, it was soon replaced by its indole counterpart 5F-MDMB-PICA, which was also widely distributed [39]. The same is true for MDMB-CHMICA, which was also detected frequently in herbal mixtures even long after its national ban at the end of 2015.

Substances that were seized less frequently by the police (e.g., 5F-ADBICA, SDB-006, and 5F-Cumyl-PINACA) were also seldom found in our post-mortem cases. Furthermore, as can be seen for Cumyl-PEGACLONE and 5F-Cumyl-PEGACLONE, the local legislative control and epidemiology of “Spice” may also have influenced the appearance of substances and their replacement by newer ones among our cases [5, 39].

Our median concentrations for various synthetic cannabinoids correlated well with concentrations found in fatal intoxication cases in literature [16, 40]. However, a considerable difference between the minimum and maximum concentrations detected for most substances was generally evident within our data (see Fig. 2). Here, several factors may have played a role. For one, first-time or occasional consumers may tolerate lower doses as opposed to chronic users, resulting in higher potentially fatal concentrations in the latter. However, from a post-mortem perspective, it is difficult to estimate the deceased’s tolerance toward a specific drug when hair analyses are not performed routinely. Furthermore, post-mortem processes, like substance degradation and redistribution and the time between consumption and death, may also influence the drug concentration in blood at the time of sampling. Our cardiac blood samples showed higher concentrations for 5F-Cumyl-PEGACLONE and MDMB-4en-PINACA compared to their peripheral blood levels, which may be a result of post-mortem redistribution. In contrast, there was no notable correlation between post-mortem interval and substance concentration in blood, at least for the five most frequently detected substances (see Fig. 2). However, these results are not representative of post-mortem cases in general. Our data include only a few cardiac blood samples, i.e., cases for which no peripheral blood was available for toxicological analysis (e.g., due to extensive post-mortem decomposition). A combination of peripheral and central blood analyses was thus not performed for any of our cases, making a direct quantitative comparison between these two sources difficult. Nevertheless, when considering central to peripheral blood ratios for synthetic cannabinoids from previously recorded cases, a lack of consistency becomes apparent [40–43]. This suggests that several other factors, such as differences in the chemical properties between substances and prior consumption patterns likely determine the degree of post-mortem redistribution of synthetic cannabinoids. Due to the rapid distribution and metabolism of synthetic cannabinoids after consumption, a longer time lapse between consumption and death may result in lower concentrations in post-mortem blood [44–46]. Overall, it remains challenging to estimate the role of a synthetic cannabinoid concentration in the death, especially with limited information about past consumption habits, as is often the case in routine post-mortem toxicology.

## Conclusions

Today, roughly one and a half decades after the appearance of the first-generation synthetic cannabinoids in “legal high” preparations, “Spice” and its effects still seem to baffle clinicians, toxicologists and legal authorities. As the number of synthetic cannabinoid receptor agonists on the market increased, their structural diversity also evolved over the years, bringing about an assortment of pharmacological profiles within this group of drugs. Nevertheless, the data provided in this publication suggest that, despite the ongoing emergence of new substances, the changes in drug laws did have a positive effect on their role in fatal intoxication cases, at least in the Munich area. In this study, we not only saw a significant drop in the total number of synthetic cannabinoid-associated fatalities since the NpSG came into force, but the causative role of these drugs in the fatalities also lost significance in recent years. Furthermore, in parallel to the legal changes, public awareness of the risks associated synthetic cannabinoid use might have been strengthened by the wide media coverage of this issue, as well as increased efforts in prevention work. Nevertheless, such cases still emerge, requiring knowledge about previous cases to assess their relevance in the fatality. Consumers of “herbal mixtures” can also never be certain of which substances are contained in such products, nor their quantity or associated health risks. It has been shown that the quantity of active ingredients between samples, and even within packages, varies significantly [47, 48], which further increases the risk for serious intoxications.

With this work, we provide a detailed report of 98 synthetic cannabinoid-related fatalities, encompassing 41 different synthetic cannabinoid drugs. All autopsies and toxicological analyses were performed at the same respective institutes, thus allowing a fair comparison between cases.

In conclusion, the limited knowledge about the pharmacokinetics, pharmacodynamics and post-mortem distribution and stability of synthetic cannabinoids still challenges forensic toxicologists in the assessment of their role in fatal intoxication cases. The availability of more detailed post-mortem case studies will undoubtedly help shed more light on the topic.

## Supplementary Information

Below is the link to the electronic supplementary material.

Supplementary file1 (DOCX 126 KB)

## Data Availability

All data generated and analyzed during this study are included in this published article and its supplementary information files.
