# Supplementary material for: “Spice”-related deaths in and around Munich, Germany: A retrospective look at the role of synthetic cannabinoid receptor agonists in our post-mortem cases over a seven-year period (2014–2020)
Source: Int J Legal Med. 2023 Apr 19;137(4):1059–69. doi: 10.1007/s00414-023-02995-2 (PMC10247575; doi:10.1007/s00414-023-02995-2)
Supplement: Supplementary file 1 — Supplementary file1 (DOCX 126 KB) [file 414_2023_2995_MOESM1_ESM.docx]

**Supplementary Information**

**“Spice”-related deaths in and around Munich, Germany:** A retrospective look at the role of synthetic cannabinoid receptor agonists in our post-mortem cases over a seven-year period (2014-2020)

International Journal of Legal Medicine

Olwen Groth*, Gabriele Roider, Verena Angerer, Jan Schäper, Matthias Graw, Frank Musshoff and Volker Auwärter

*Corresponding author:

E-Mail: [olwen.groth@med.uni-muenchen.de](mailto:olwen.groth@med.uni-muenchen.de)

Affiliation: Institute of Forensic Medicine, University of Munich, 80336 Munich, Germany

**Table S1.** Summary of cases with a toxicological significance score of 3, for which synthetic cannabinoids could be detected in femoral venous blood.

| Toxicological Significance Score 3 (TSS 3) | | | | | | | | | |
| --- | --- | --- | --- | --- | --- | --- | --- | --- | --- |
| # | Synthetic cannabinoids detected, including the approximate concen­trat­ions in femoral venous blood [µg/L] | | Sex  and  Age | Case  history | Other substances detected in femoral venous blood [µg/L] | Blood alcohol concentration (bac) in femoral venous blood  Alcohol concentration in urine (uac) [g/100 mL] | Main  cause  of death | Most relevant  findings at  autopsy | PMI* |
| **1** | 5F-ADB | 14 | M 23 | Found in a comatose state in the bathroom stall of a fast-food restaurant; Drug paraphernalia were found next to the body; Released 2 days prior after 11 months of imprisonment; Died, despite an emergency physician’s efforts to resuscitate | 6-Monoacetylmorphine (4,3); Morphine (120); Codeine (6,6); Noscapine (21); Papaverine (1,3); Para­cet­a­mol (450); Naloxone (3,5) | BAC: 0,00  UAC: 0,01 | Polydrug intoxication  with heroin and  5F-ADB | Macromorphological signs  of increased intracranial pressure; Bloody pulmonary  oedema | 20 hrs |
| **2** | 5F-ADB | 0,65 | M 59 | Found dead in his apart­ment, lying in his own vomit | THC (traces);  THC-OH (traces);  THC-COOH (3,4) | BAC: 0,40  UAC: 0,52 | Aspiration of vomit due to central nervous system depression by 5F-ADB and alcohol | Significant, acute increase in brain volume; No signs of a fatty liver; Large amount of vomit in the airways | 2-6  days |
| **3** | 5F-ADB | 0,54 | F 36 | Known history of heroin abuse;  Found dead approximately  2 hours after she was  last seen alive | Levetiracetam (5100) | BAC: 3,12  UAC: 4,52  Significantly elevated levels of methanol in blood and urine | Intoxication with  5F-ADB  and alcohol | Significant pulmonary oedema; Increased brain volume, with signs of acute intracranial pressure; Pronounced alcoholic, aromatic odour streaming from the abdominal cavity | 20 hrs |
|  | 5F-Cumyl-  PEGACLONE | < 0,1 |  |  |  |  |  |  |  |
| **4** | 5F-ADB | 0,47 | M 30 | Found dead after jumping or falling from his 3^rd^ story apartment; Known history of drug abuse | Paracetamol (traces) | BAC: 0,01  UAC: 0,01 | Traumatic brain injury after jumping/falling from a 3^rd^ story balcony; Synthetic cannabinoids may have led to psychosis or a panic attack, causing the deceased to jump/fall | Pronounced disintegration of the face, forehead, cal­va­ria and frontal cranial base; Cerebral artery avul­sion; Complete avulsion of the thoracic aorta;  Thoracic spine fractures | 2 days |
|  | MDMB-CHMICA | 0,24 |  |  |  |  |  |  |  |
| **5** | 5F-ADB | 0,43 | M 25 | Known history of drug abuse; Found dead in a friend’s apartment | Mirtazapine (26); Hydro­morphone (5,1); Pregabalin (2100), 4-Methyl­amino­antipyrine (*not quantified*) | BAC: ~ 1  UAC: 1,49 | Intoxication with  synthetic cannabinoids  and alcohol | Clear signs of increased intra­cranial pressure; Aspiration of stomach contents; Organs showed beginning signs of post-mortem decomposition | 4 days |
|  | 5F-Cumyl-P7AICA | 4,0 |  |  |  |  |  |  |  |
| **6** | 5F-ADB | 0,42 | M 38 | Long history of drug and alcohol abuse; Found dead in his apartment; A “bong” was found next to the body | Pregabalin (traces) | BAC: 2,30  UAC: ~ 3,0  Elevated levels of methanol, acetone and 2-propanol in blood and urine | Intoxication with  5F-ADB  and alcohol | Advanced state of decomposition  and autolysis; Fruity, aromatic odour seeping from the abdominal cavity; Fatty liver | 10 days |
| **7** | 5F-ADB | 0,38 | M 30 | Went to bed shortly after consuming “herbal mixtures”; Found in a comatose state approximately 30 minutes later; Died, despite the rescue service’s efforts to resuscitate | - | BAC: 3,10  UAC: 4,31  Significantly elevated levels of methanol in blood and urine | Intoxication with  5F-ADB and alcohol | Hyperaemia of the inner organs; Slight cerebral oedema; Full bladder; Aromatic odour seeping from the abdominal cavity | 16 hours |
| **8** | 5F-ADB | 0,21 | M 36 | Found dead in his apartment, with a possible injection site in the groin; Unknown tablets and drug paraphernalia, incl. a glass pipe and disposable syringes, were found in the room; Long history of drug consumption (*e.g.* heroin, “bath salts”); Suffered from, panic attacks and epilepsy; Was pre­scribed lorazepam, oxaze­pam and clonazepam by his physician | Morphine (55); Nor­morphine (*not quantified*); Diazepam (290); Nordazepam (340); Oxa­zepam (41); Tema­zepam (26); Lora­zepam (43); Pre­ga­ba­lin (3100); Buprenorphine (2,5); Norbuprenorphine (1,2); Paracetamol (traces) | BAC: 0,09 | Aspiration of gastric contents due to an intoxication with synthetic cannabinoids and the other central nervous system depressants detected in blood | Gastric contents in the upper airways, reaching up to the subsegmental bronchi; Full stomach;  Increased brain volume, with signs of acute  intracranial pressure | 1-2 days |
|  | Cumyl-PEGACLONE | 0,84 |  |  |  |  |  |  |  |
|  | 5F-PB-22 | 0,18 |  |  |  |  |  |  |  |
|  | EG-018 | 0,14 |  |  |  |  |  |  |  |
| **9** | 5F-ADB | 0,19 | M 37 | Found dead approximately  5 hrs after consuming  “Spice” and alcohol  with friends | Doxepin (7,9);  Nordoxepin (22); Paracetamol (21) | BAC 2,20  UAC 2,82  Elevated concentrations of methanol in urine | Aspiration of gastric contents due to an intoxication with  5F-ADB and  alcohol | Gastric contents, reaching deep into the airways; Pronounced abnormal distention of both lungs; Signs of acute intracranial pressure | 16 hours |
| **10** | A 5F-ADB  hydrolysis product | *Not quant.* | M 50 | Found dead in his  apartment, together with “herbal mixtures” and  drug paraphernalia | 6-Monoacetalmorphine (7,6); Morphine (200); Codeine (15); Methadone (200); EDDP (19); Pregabalin (23 000); 7-Aminoclonazepam (1,8); Paracetamol (92) | BAC: 0,01  UAC: 0,03 | Intoxication with heroin,  in combination with methadone and  MDMB-4en-PINACA | Increased brain volume with signs of mostly infratentorial intracranial pressure; Haemorrhagic pulmonary oedema; Two fresh injection sites in the crook of the right arm; 130 mL of urine in the bladder | 1 day |
|  | MDMB-4en-PINACA | 0,99 |  |  |  |  |  |  |  |
|  | 4F-MDMB-BICA | *Not quant.* |  |  |  |  |  |  |  |
|  | 4F-MDMB-BINACA | < 0,1 |  |  |  |  |  |  |  |
|  | 5F-MDMB-PICA | < 0,1 |  |  |  |  |  |  |  |
| **11** | 5F-MDMB-PICA | 0,88 | M 44 | Found dead  in his apartment;  Known history of drug  and alcohol abuse | Lorazepam (35); Mir­tazapine (3,9); Quetiapine (2,9); Sertraline (37); Levetiracetam (830) | BAC: ~ 2,2  UAC: 2,97 | Intoxication with  synthetic cannabinoids  and alcohol | Significant internal post-mortem decomposition; Fatty liver; Full bladder; Little gastric content | 6 days |
|  | MDMB-FUBICA | < 0,1 |  |  |  |  |  |  |  |
|  | Cumyl-4CN-BINACA | *Not quant.* |  |  |  |  |  |  |  |
| **12** | 5F-MDMB-PICA | 7,2 | M 43 | Known history of drug abuse; The deceased could not be reached for 4 weeks by relatives, after which he was found dead in his apartment; A “bong”, tabaco mixture and drug paraphernalia were discovered | Tilidine (93);  Nortilidine (200);  Naloxone (< 1,0) | BAC: ~ 1,5 | Intoxication with  5F-MDMB-PICA,  possibly combined  with tilidine  and alcohol | Significant external and internal post-mortem decomposition; Brown-green coloured stomach contents, with a white, clumpy residue | 4 weeks |
| **13** | 5F-MDMB-PICA | 3,2 | M 45 | Known history of drug abuse; The deceased started seizing and fell unconscious, after which he was trans­por­ted to hospital; Diagnosis: hyper­kalaemia, opisthotonos and muscle rigor; The patient died 2 hrs after hospital admission | Cocaine (traces);  Ben­zoyl­ecgonine (traces);  Ec­gonine methyl ester (1,0); Lidocaine (*not quantified*); Rocuronium (*not quantified*); Furosemide (*not quantified*) | BAC: 0,02  UAC: 0,04 | Intoxication with  5F-MDMB-PICA | Signs of intracranial pressure; General atherosclerosis;  Atherosclerosis of the coronary artery (not pronounced); No significant findings of the liver,  spleen or pancreas | 19 hours |
| **14** | 5F-MDMB-PICA | 0,64 | M 46 | Found dead in his apartment, with 2 packages containing “herbal mixtures” and “joints” | Pregabalin (2400) | BAC: 0,02 | Intoxication with  5F-MDMB-PICA | Onset of post-mortem decomposition and autolysis; Significant coronary sclerosis | 3 days |
| **15** | AB-CHMINACA | 28 | M 16 | Known history of “herbal mixture” and “bath salt” con­sump­tion in increasing amounts; Found dead on a playground in the early morning hours, with a hookah and tobacco next to the body; Added “a white powder” and tobacco to the hookah the previous night and smoked it | - | BAC: 0,05  UAC: 0,47 | Intoxication with  AB-CHMINACA, which either led to cardiac arrhythmias and/or asphyxiation after the aspiration of stomach contents | Significant cardiac hypertrophy;  Fluid in the pericardium; Gastric contents in the airways; Significant intracranial pressure | 1-2 days |
| **16** | AB-CHMINACA | 15 | M 32 | Known history of drug abuse; Found in a comatose state in the apartment of a friend, after having consumed alcohol together; The emergency physician’s attempts to reanimate were unsuccessful | Nordazepam (13); Lorazepam (8,8); Doxepin (8,9); Nor­doxepin (29); Naloxone (3,2); Lidocaine (traces) | BAC: 2,35  UAC: 3,40 | Fatal intoxication with  AB-CHMINACA and alcohol | Signs of increased intra­cranial pressure; Pronounced liver cirrhosis; Pronounced haemorrhagic pulmonary oedema; Enlarged spleen | 19 hours |
| 17 | AB-CHMINACA | 12 | M 35 | Found dead in his apartment; Known history of drug abuse;  Drug paraphernalia were found  at the scene | Tramadol (140);  N-Desmethyltramadol (13); O-Desmethyl­tramadol (120); Buprenorphine (1,7); Nor­buprenorphine (0,28) | BAC: 0,00  UAC: 0,05 | Most likely an intoxication with AB-CHMINACA in combination with the other central nervous system depressants | Fresh injection site on the right arm; Stomach contents in the upper and lower airways, however without reaching the lungs | 3 days |
| **18** | AB-CHMINACA | 8,2 | M 27 | Known history of heroin consumption; Found dead in a friend’s apartment; Heroin and drug paraphernalia were found at the scene | Pipamperone (13); Bupropion (990); Buprenorphine (6,5); Nor­buprenorphine (2,8); Methylphenidate (22); Ritalinic acid (450) | BAC: 0,05  UAC: 0,15 | Intoxication with buprenorphine and  AB-CHMINACA, possibly also methylphenidate  and bupropion | Significant post-mortem decomposition; Distinct signs of intracranial pressure; Blood congestion in the organs; Several fresh needle puncture sites on both arms | 1 day |
| **19** | AB-CHMINACA | 4,2 | M 30 | Known history of drug abuse  (e.g. synthetic cannabinoids); The deceased was treated for insomnia, anxiety and ADHS; Collapsed in the bathroom; Unsuccessful resuscitation by  the emergency physician | - | BAC: 0,00 | Intoxication with  AB-CHMINACA | Pronounced acute increase  of brain volume;  Significant atheromatosis of the coronary artery;  Cardiac hypertrophy | 15 hours |
| **20** | AB-CHMINACA | 3,7 | M 35 | The decomposed body of the deceased was found in his apartment;  Known history of drug abuse | - | BAC: 0,6  UAC: 1,09 | Asphyxiation due to aspiration of stomach contents after an intoxication with AB-CHMINACA | Stomach contents in the inner airways; The inner organs were in an advanced state of decomposition | 1 week |
| **21** | AB-CHMINACA | 3,3 | M 36 | Consumed “research chemicals” with his girlfriend; Stayed awake the whole night, after which both fell asleep in the early morning hours; Found dead in the evening; E-Liquids were found at the scene | Amphetamine (5,8) | BAC: 0,05  UAC: 0,06 | Intoxication with  AB-CHMINACA | Significant post-mortem decomposition of the inner organs; Haemorrhagic pulmonary oedema; Moderate intracranial pressure | 17 hours |
| **22** | AB-CHMINACA | 2,6 | M 63 | Found dead in a friend’s apartment after a party | Quetiapine (22); Morphine (1,4); Risperidone (3,1) | BAC: 0,00  UAC: 0,00 | Most likely the cardiotoxic effects of AB-CHMINACA combined with a history of heart disease | Significant cardiac hypertrophy (600 g) and pulmonary oedema; Cerebral oedema | 2 days |
| **23** | AB-CHMINACA | 0,2 | M 45 | Known history of drug abuse (e.g. substances from the internet); Underwent psychiatric treatment for schizophrenia; Found dead in his apartment, with drug para­phernalia, melperone, risperidone, prothipendyl and two bags with “2-FA” and “MDMB-CHMINACA” | Melperone (46);  Promethazine (14);  Risperidone (7,3);  4-Fluoroamphetamine (430); 2-Fluo­ro­amphetamine (57) | BAC: 0,12  UAC: 0,24 | Intoxication with  4-fluoroamphetamine  and ADB-CHMINACA | Several injection sites on both arms;  Signs of intracranial pressure; Aspiration of gastric contents | 1 day |
|  | ADB-CHMINACA | 22 |  |  |  |  |  |  |  |
| **24** | MDMB-CHMICA | 1,8 | M 30 | Found dead in his apartment with “herbal mixtures”, labelled MMB-CHMINACA (= MDMBCHMICA), an E-cigarette with several E-liquids, a glass “bong” and an empty bottle of Vodka; Was recently released from a  drug detoxification programme | Mirtazapine (710); Duloxetine (130); Amphetamine (3,9) | BAC: 1,20  UAC: ~ 1,4  Slightly elevated levels of methanol in  blood and urine | Intoxication with synthetic cannabinoids, in combination with  alcohol and  mirtazapine | No clearly defined injection sites found; Hyperaemia of the inner organs; Abnormal distention of the lungs; Gastric contents in the airways; Cerebral oedema, with signs of increased intracranial pressure | 21 hours |
|  | AB-FUBINACA | 8,4 |  |  |  |  |  |  |  |
| **25** | MDMB-CHMICA | 1,5 | M 32 | Known history of drug abuse; Found dead in his mother’s basement; “Herbal mixtures” were found close to the body | Fentanyl (15);  Norfentanyl (3,3) | BAC: 0,05 | Intoxication with fentanyl, possibly also  MDMB-CHMICA | Increased brain volume, with signs of intracranial pressure; Hyperaemia of the inner organs | 3 days |
|  | SDB-006 | *Not quant.* |  |  |  |  |  |  |  |
| **26** | MDMB-CHMICA | 0,33 | M 38 | Found dead in his apartment; Known history of drug and alcohol abuse; Adipositas per magna | - | BAC: 1,68 | Intoxication with  MDMB-CHMICA and alcohol, leading to fatal cardiotoxicity and/or asphyxiation after aspiring stomach contents | Extremely fatty liver; Gastric contents in the airways;  Significant cardiac hypertrophy (680 g) | 1 day |
| **27** | ADB-CHMINACA | 6,9 | M 32 | Found dead in a friend’s apartment, with drug para­phernalia, including “bongs”, Marihuana and syringes; Known history of drug abuse | Tapentadol (150) | BAC: 1,61  UAC: 2,65 | Intoxication with  ADB-CHMINACA, tapentadol and alcohol | Signs of intracranial pressure (weight of the brain: 1740 g); Pulmonary oedema;  400 mL of urine in the bladder | 1 day |
| **28** | ADB-CHMINACA | 6,8 | M 20 | Found in a comatose state in his apartment; The emer­gen­cy physician’s attempt to resuscitate the patient was unsuccessful; A container with a white powder was found at the scene | - | BAC: 0,42  UAC: 1,42 | Intoxication with  ADB-CHMINACA, possibly combined  with alcohol | Increased brain volume, with signs of intracranial pressure; Acute hyperaemia of the inner organs;  Significant lung oedema | 1 day |
| **29** | ADB-CHMINACA | 6,2 | M 25 | Found dead in his apartment, with two packets, containing a white powder; Long history of drug abuse; Was treated for schizophrenia | Citalopram (67);  4-Fluoroamphetamine (traces) | BAC: 0,02 | Most likely an intoxication with ADB-CHMINACA | Injection site found on  the left arm;  Cerebral oedema;  Fatty liver | 2 days |
| **30** | ADB-CHMINACA | 5,3 | M 20 | Found in a comatose state in a friend’s apartment; Drugs, “herbal mixtures” and alcohol were consumed the night before | - | BAC: 1,42  UAC: 2,98 | Intoxication with  ADB-CHMINACA  and alcohol | Moderately filled bladder; Little content in the gastro­intestinal tract; Signs of moderate intracranial pressure | 3 days |
| **31** | ADB-CHMINACA | 4,0 | M 33 | Found dead in the room of a psychiatric institution, with aripiprazole, meto­clo­p­ra­mide, sertraline, trimipra­mine, levo­methadone, pan­to­pra­zole and a packet con­tai­ning a white powder; Known history of drug abuse | Trimipramine (1000);  Sertraline (570); Metha­done (600); EDDP (30);  Levo­mepromazine (54);  Aripiprazole (*not quantified*);  Metoclopramide (190) | BAC: 0,01  UAC: 0,01 | Intoxication with  ADB-CHMINACA, possibly combined with the other drugs detected in blood  and myocarditis | Haemorrhagic pulmonary oedema; Pronounced fatty liver; Yellowish, slimy content in the airways | 3 days |
| **32** | ADB-CHMINACA | 1,2 | M 34 | Known history of drug abuse; Underwent drug withdrawal therapy approximately 1 week prior to death; Col­lap­sed in a friend’s apartment; Emergency physician de­clared him dead after an unsuccessful attempt to resuscitate | 9-Hydroxyrisperidone (traces); Lidocaine (*not quantified*); Amphetamine (4,4) | BAC: 0,02  UAC: 0,04 | Most likely an  intoxication with  ADB-CHMINACA | Expansion of the right heart chamber; Fluid and blood-filled lungs; Hyperaemia of the liver and spleen | 14 hours |
| **33** | ADB-CHMINACA | 0,88 | M 34 | Found dead in his room, with a “bong” in his hand; Consumed cannabis and “herbal mixtures” in the past; Vomit was found at the scene | - | BAC: 2,92  UAC: 0,88 | Intoxication with  ADB-CHMINACA  and alcohol | Acute hyperaemia of the inner organs; Pronounced pulmonary oedema; Increased brain volume, with signs of acute intracranial pressure | 2 days |
| **34** | AB-FUBINACA | 4,5 | M 23 | Found dead in his room, next to the bed; Known history of drug abuse (cannabis); Doxepin, mirtazapine and phenibut were found at the scene | Doxepin (9,2); Mirtazapine (99); Citalopram (14); Lormetazepam (traces); Methiopropamine (very high concentration) | BAC: Traces  UAC: Traces | Intoxication with  synthetic cannabinoids and methiopropamine | Blood congestion in the organs; Pronounced haemorrhagic pulmonary oedema | 1-2 days |
|  | BB-22 | 2,3 |  |  |  |  |  |  |  |
|  | APINACA | 54 |  |  |  |  |  |  |  |
|  | 5F-APINACA | 55 |  |  |  |  |  |  |  |
|  | APICA | < 0,1 |  |  |  |  |  |  |  |
|  | THJ-2201 | 0,1 |  |  |  |  |  |  |  |
|  | 5F-PB-22 | 0,46 |  |  |  |  |  |  |  |
| **35** | 4F-MDMB-BINACA | 19 | M 37 | Found dead outside his apartment building; Presumably jumped / fell from his 8^th^ story apartment balcony | Norbuprenorphine (1,4) | BAC: 0,00 | Bled to death after an aorta rupture, following a jump/fall from a significant height; The prior consumption of  4F-MDMB-BINACA may have caused psychosis or a state of anxiety, which may have led to the incident | Very pale organs;  Complete aortic rupture, with significant bleeding;  Rib fractures;  Tibial shaft fracture; Significant right cardiac hypertrophy | 2 days |
| **36** | 5F-PB-22 | < 0,1 | M 26 | Admitted to hospital after consuming “herbal mixtures”; Died soon after | Lidocaine (*not quantified*) | BAC: 0,00  UAC: 0,00 | Intoxication with  synthetic cannabinoids | Haemorrhagic pulmonary oedema; Acute increased brain volume | 1-2 days |
|  | AB-PINACA | 0,47 |  |  |  |  |  |  |  |
| **37** | ADB-PINACA | 41 | M 44 | Found dead approximately 6 hours after consuming “herbal mixtures” with friends; “Herbal mixtures” with the following inscriptions were found at the scene: “Twilite, Passion Sense, Cherry” | - | BAC: 1,97  UAC: 2,56  Elevated levels of methanol in  blood and urine | Intoxication with  ADB-PINACA  and alcohol | Increased brain volume with signs of intracranial pressure; Acute hyperaemia of the inner organs; Pronounced aromatic odour coming from the abdominal cavity, but not from the stomach contents | 11 hours |
| **38** | MDMB-4en-PINACA | 0,31 | M 38 | Found in a comatose state in his bed; Emergency physician was not able to revive him; Known history of drug abuse (“Spice”, “bath salts”, pregabalin, heroin); Underwent drug substitution therapy with buprenorphine | Methadone (1000); EDDP (85); Morphine (traces); Bro­ma­zepam (42); Hydroxy­bro­ma­zepam (*not quantified*); Lo­razepam (4,8); Pregabalin (6100); Gabapentin (320); Mir­tazapine (traces); Bu­pro­pi­on (56); Methyl­phenidate (17); Ritalinic acid (190); α-PiHP (11) | BAC: 0,11  UAC: 0,14 | Intoxication with methadone and MDMB-4en-PINACA, possibly also with pregabalin and the other central nervous system depressants | Significant haemorrhagic pulmonary oedema; Increased brain volume, with signs of intracranial pressure; Moderately fatty liver; Little urine in the bladder; Several scars in the groin region | 29 hours |
|  | 4F-MDMB-BICA | *Not quant.* |  |  |  |  |  |  |  |
| **39** | THJ-2201 | 0,50 | M 27 | The deceased celebrated a party with friends, during which he presumably con­sumed large amounts of al­co­hol; The following day, he was found dead in his apart­ment, with a large amount of blood exiting his mouth | - | BAC: 0,76  UAC: 0,98 | Intoxication with THJ-2201 in combination with alcohol | Increased brain volume, with signs of acute - mainly supratentorial - intracranial pressure; Pronounced pulmonary oedema of the right lobe; Splenomegaly | 20 hours |
| **40** | STS-135 | 0,10 | M 28 | The deceased smoked a “joint”, containing “Spice” and soon after complained of tachycardia; He went to bed around midnight and was found dead early the next morning | - | BAC: 0,00  UAC: 0,10 | Most likely an intoxication with synthetic cannabinoids | Swollen brain, with signs of intracranial pressure | 2-3 days |
|  | EAM-2201 | 0,32 |  |  |  |  |  |  |  |
|  | JWH-210 | 0,54 |  |  |  |  |  |  |  |
| **41** | JWH-122 | 25 | M 29 | Found dead in his apartment with Marihuana, LSD, heroin, etizolam, clonazo­lam, dicla­ze­pam, O-PCE, butylone, 2-FMA, ethyl­phenidate, *N*-ethyl­hexedrone and AMT; Known history of drug abuse | γ-Hydroxybutyric acid (GHB, 700 000); Quetiapine (630);  Ritalinic acid (traces);  Eti­zo­lam (11); Ethyl­phenidate (1,5); 2F-Amphetamine (2,4) | BAC: ~ 1,2  Elevated levels of methanol in blood | Intoxication with GHB or precursor drugs, combined with JWH-122 and other central nervous system depressants | Significant post-mortem decomposition, with partial mumification and maggot infestation; Full stomach | 32 days |

*Post-mortem interval = Estimated delay between death and sampling of biological specimens

**Table S2.** Summary of cases with a toxicological significance score of 2, for which synthetic cannabinoids could be detected in femoral venous blood.

| Toxicological Significance Score 2 (TSS 2) | | | | | | | | | | |
| --- | --- | --- | --- | --- | --- | --- | --- | --- | --- | --- |
| # | Synthetic cannabinoids detected in femoral venous blood, including their approximate concentrations  [µg/L] | | Sex  Age | Case  history | Other substances detected in femoral venous blood (µg/L) | Blood alcohol concentration (bac) in femoral venous blood  Urine alcohol concentration (uac)  [g/100 mL] | Main cause of death | Most relevant  findings at autopsy | PMI* |  |
| 42 | 5F-ADB | 0,29 | M 49 | The deceased was admitted to hospital soon after complaining about difficulty breathing; He died 80 minutes later; Known history of drug abuse; Marihuana and pregabalin were found in the deceased’s apartment | Pregabalin (70) | BAC: 0,01  UAC: 0,00 | The deceased most likely died of the cardiotoxic effects of  5F-ADB | Massive coronary atherosclerosis, with complete block of the right coronary artery; Massive cardiac hypertrophy (heart weight: 660 g); Signs of an infarction on the walls of the right heart chamber; Massive pulmonary oedema both sides | 2 days |  |
|  | An AB-FUBINACA or AMB-FUBINACA metabolite | *Not quant.* |  |  |  |  |  |  |  |  |
| 43 | 5F-ADB | 0,27 | M 35 | Suffered from alcoholism, asthma and depression; Found dead by his friends the day after consuming alcohol together | THC (2,0); THC-OH (1,4); THC-COOH (12); Cannabinol (*not quantified*) | BAC: 4,83  UAC: 5,87 | Alcohol intoxication, possibly in combination with synthetic cannabinoids | Increase of brain volume, without signs of intracranial pressure; Fatty liver; Acute hyperaemia of the inner organs; Aromatic smell exiting the abdominal cavity | 1-2 days |  |
|  | NE-CHMIMO | 1,00 |  |  |  |  |  |  |  |  |
|  | MDMB-CHMICA | 0,13 |  |  |  |  |  |  |  |  |
| 44 | 5F-ADB | 0,22 | M 32 | Found dead in a friend’s apartment, History of excessive alcohol consumption | 6-Monoacetylmorphine (< 1,0); Morphine (99); Codeine (12); Noscapine (1,1); Papaverine (< 1,0); Nordazepam (7,7); Paracetamol (360); Ibuprofen (7700) | BAC ~ 1,0  UAC 2,75  Elevated concentrations of methanol in blood and urine | Fatal intoxication with heroin, likely combined with alcohol and  5F-ADB | Signs of pronounced intracranial pressure and increased brain volume (brain weight: 1752 g); Pulmonary oedema; Fruity, aromatic odour streaming from the abdominal cavity; Full bladder | 1-2 days |  |
| 45 | 5F-ADB | 0,10 | M 40 | Known history of  alcohol abuse;  Found dead in the  botanical gardens | - | BAC: 3,37  UAC: 4,35  Significantly elevated levels of methanol, acetone and 2-propanol in  blood and urine | Intoxication with alcohol and 5F-ADB, in combination with an underlying heart disease | Signs of significant intracranial pressure; Alcoholic, aromatic odour streaming from the abdominal cavity; Enlarged heart; Moderately fatty liver; Enlarged spleen; Little stomach contents; Little urine in the bladder | 1-2 days |  |
| 46 | 5F-ADB | < 0,1 | M 56 | Known history of drug abuse (*e.g.* synthetic opioids); History of hypertension; Found dead in his apartment one day after he was last seen alive | Fluoxetine (290); Trimi­pramine (680); Sildenafil (42); Amlodipine (4,3); Lido­caine (traces); Diphen­hydramine (99); Mitragynine (8,4); U-47700 (very high concentration); 3,4-DMMC (1,8); Butylone (3,5) | BAC 0,02  UAC 0,03 | Intoxication with U-47700 and ADB-FUBINACA | Pronounced haemorrhagic pulmonary oedema; Pronounced atheromatosis of the coronary artery; Fatty liver; Onset of internal post-mortem decomposition | 2 days |  |
|  | ADB-FUBINACA | 4,7 |  |  |  |  |  |  |  |  |
|  | AM-2201 | 0,12 |  |  |  |  |  |  |  |  |
|  | AMB-CHMICA | < 0,1 |  |  |  |  |  |  |  |  |
|  | EG-018 | 4,7 |  |  |  |  |  |  |  |  |
| 47 | 5F-ADB | < 0,1 | M 39 | Found dead in his apartment, with an “herbal mixture” (“Bonzai”) lying next to the body; Known history of drug abuse | - | BAC: 0,01  UAC: 0,03 | Considering the deceased’s underlying heart condition, his death was likely caused by the cardiotoxic effects of  5F-Cumyl-PEGACLONE | Cardiac hypertrophy; Signs of a heart attack on the septum; Pulmonary oedema both sides; Aspiration of gastric contents; Fatty liver | 2 days |  |
|  | 5F-Cumyl-PEGACLONE | 0,33 |  |  |  |  |  |  |  |  |
| 48 | 5F-MDMB-PICA | 1,2 | M 51 | Tripped and fell, after which he remained lying dead on the kitchen floor; The deceased’s wife waited 2 days before informing the authorities; Known history of alcohol abuse and consumption of kratom | Mitragynine (130); 7-Hydroxy­mitragynine (30); Bromazepam (traces); Diaze­pam (86); Nor­dazepam (130); Oxazepam (4,8); Temazepam (5,7); Lidocaine (traces); Amfe­pramone (5,6); Eth­cathinone (12); Nor(-pseudo-)­ephedrine (8,4) | BAC: 0,01  UAC: 0,85  Slightly elevated  methanol levels  in blood and urine | Most likely an intoxication with 5F-MDMB-PICA,  in combination with mitragynine and alcohol | Advanced state of post-mortem decomposition; Signs of increased intracranial pressure; Moderately filled gastro­intestinal tract; No significant findings in the heart | 3 days |  |
| 49 | 5F-MDMB-PICA | 0,89 | M 24 | Found in a comatose state in the bathroom stall of a fast-food restaurant; Drug paraphernalia were found at the scene; Known history of drug abuse; Declared dead by the emergency physician | 6-Monoacetylmorphine (4,3); Morphine (370); Codeine (36); Normorphine; Norcodeine; Noscapine; Papaverine (*not quantified*);  Buprenorphine (0,57); 7-Amino­clonazepam (11); Paracetamol (2200) | BAC: 0,01  UAC: 0,18 | Intoxication with heroin, possibly in combination with buprenorphine and  5F-MDMB-PICA | Lesions on the left arm, that may possibly be injection sites; Reddish, pulmonary oedema, with foam reaching into the branches of the airways and mouth | 2-3 days |  |
| 50 | 5F-MDMB-PICA | 0,70 | M 23 | History of drug abuse, Envelope with a white powder (U-47700), used “joints” and an empty packet of “herbal mixture” (Gold Extreme) were found in the deceased’s room; Died despite reanimation efforts | U-47700 (490);  Lidocaine (*not quantified*) | BAC: 0,01  UAC: 0,01 | Polydrug intoxication with  U-47700 and  synthetic cannabinoids | Cardiomegaly (heart weight: 548 g); Cardiac hyper­trophy; Haemorrhagic pulmonary oedema; Terminal cerebral oedema | 19 hours |  |
|  | 5F-ADBICA | 0,14 |  |  |  |  |  |  |  |  |
|  | AMB-CHMICA | < 0,1 |  |  |  |  |  |  |  |  |
|  | MDMB-CHMICA | < 0,1 |  |  |  |  |  |  |  |  |
|  | MDMB-FUBICA | < 0,1 |  |  |  |  |  |  |  |  |
| 51 | 5F-MDMB-PICA | 0,16 | F 29 | The deceased was released from prison shortly before the incident; Long history of drug abuse; Found in a comatose state after consuming heroin, alcohol and “herbal mixtures”; Emergency physician resuscitated; Declared dead approximately 1 hr later; Drug paraphernalia were found at the scene | 6-Monoacetylmorphine (13); Morphine (370);  Codeine (32);  Oxazepam (570); Pregabalin (6800); Paracetamol (620); Dextromethorphan (7,8); Lidocaine (*not quantified*) | BAC: 0,00  UAC: 0,03 | Intoxication with heroin  and 5F-MDMB-PICA | Signs of intracranial pressure; Fresh injection sites in the crook of the left arm | 12 hours |  |
|  | ADB-PINACA | 0,72 |  |  |  |  |  |  |  |  |
| 52 | 5F-MDMB-PICA | 0,13 | M 32 | Found in a comatose state in bed by his girlfriend, after which he was transported to hospital; Prior consumption of lorazepam and pregabalin; Declared dead approximately 1,5 hours later; Known history of drug abuse | Methadone (130); EDDP (17); 7-Amino­clonazepam (1,4); Diazepam (14); Nor­dazepam (10); Oxazepam (traces); Temazepam (traces); Lorazepam (21); Pregabalin (8500); Mir­tazapine (22); 4-Methyl­amino­antipyrine (*not quant*); Paracetamol (traces); Methyl­phenidate (0,4); Ritalinic acid (6,0); Lido­caine (2,6); Naloxone (> 50) | BAC: 0,33  UAC: 0,57 | Aspiration of gastric contents after an intoxication with central nervous system depressing drugs | Significant signs of post-mortem decomposition and autolysis;  Signs that massive aspiration of gastric contents had occurred; Bloody pulmonary oedema; Hyper­aemia of the inner organs; No significant atherosclerosis;  Several older injection sites in the crook of the right arm | 1 day |  |
|  | EG-018 | 2,3 |  |  |  |  |  |  |  |  |
| 53 | 5F-MDMB-PICA | < 0,1 | M 29 | Long history of drug abuse (mostly heroin), Found in a comatose state approx. 20 minutes after consuming “herbal mixtures”; Emergency physician’s efforts to resuscitate were unsuccessful | 6-Monoacetylmorphine (18); Morphine (270); Codeine (27); Diaze­pam (12); Nordazepam (12); Lora­ze­pam (9,7); Alprazolam (2,8);  Quetiapine (traces); Para­cet­a­mol (1000); Lidocaine (traces); | BAC: 1,65  UAC: 2,33 | Intoxication with heroin, possibly in combination with ADB-PINACA  and alcohol | Several injection sites in the groin area; Pronounced haemorrhagic pulmonary oedema; Intracranial pressure | 16 hours |  |
|  | ADB-CHMINACA | 0,26 |  |  |  |  |  |  |  |  |
|  | ADB-PINACA | 7,0 |  |  |  |  |  |  |  |  |
| 54 | AB-CHMINACA | 0,24 | M 50 | Long history of drug abuse; The body was discovered in an advanced state of decomposition in the deceased’s apartment; Drug paraphernalia, a cannabis plant, amitriptyline and citalopram were found | Morphine (41); Codeine (4,3); Citalopram (660); Nor­triptyline (traces); Pa­ra­cet­amol (200); THC (high concentration); THC-OH (*not quantified*); THC-COOH (*not quantified*) | BAC: 0,45  UAC: 0,72 | Intoxication with morphine derivatives like heroin, in combination with  AB-CHMINACA | Advanced external and internal post-mortem decomposition; 20 mL of urine in the bladder; Brain completely diffluent | 11 days |  |
| 55 | MDMB-CHMICA | 0,1 | M 60 | Was prescribed tramadol and metamizole for backache; Found dead in his apartment the following day; Known history of drug abuse | Tramadol (400); N-Des­methyl­tramadol (150); O-Desmethyl­tramadol (120); 4-Methylamino­antipyrine (*not quantified*); Methyl­meth­cathinone (116) | BAC: 0,00  UAC: 0,00 | The consumption of cathinone derivatives likely led to hyperthermia, vomiting, electrolyte imbalance and cardiac arrhythmias | Older injection sites on both arms; Signs of intracranial pressure; Pulmonary oedema; Fatty liver | 1 day |  |
| 56 | MDMB-CHMICA | 2,3 | M 25 | Found dead in the sitting room two days after locking himself in; A belt was tied around the deceased’s neck; Sex toys, contaminated with faeces, as well as “herbal mixtures”, “Poppers” and a white powder were discovered | Methylone (49); Ethylphenidate (64); Ritalinic acid (520); Lidocaine (*not quantified*); Methiopropamine (760) | BAC: 0,00  UAC: 0,11 | Intoxication with mainly methiopropamine, but also the other psychoactive substances detected in femoral venous blood | Sticky slime in the airways; Punctate bleeding in the eyes; Slight cardiac hypertrophy | 3 days |  |
|  | 5F-AMB | 0,22 |  |  |  |  |  |  |  |  |
|  | STS-135 | < 0,1 |  |  |  |  |  |  |  |  |
| 57 | 5F-Cumyl-PEGACLONE | 0,25 | F 32 | Long history of drug abuse (*e.g.* “herbal mixtures”, heroin); The deceased was declared missing; She was found in the attic of her own home 11 days after disappearing; A packet of an ”herbal mixture” was found next to the body | 6-Monoacetylmorphine (39); Morphine (730); Nor­morphine (*not quantified*); Codeine (579, Noscapine (> 50); Papaverine (29); Paracetamol (73); Benzoylecgonine (traces); Ecgonine methyl ester (1,5); Dibutylone (< 0,5) | BAC: 0,02  UAC: 0,04 | Fatal intoxication with heroin and  4F-MDMB-BINACA | Onset of post-mortem decomposition; Signs of intracranial pressure | 16 days |  |
|  | 4F-MDMB-BINACA | *Not quant.* |  |  |  |  |  |  |  |  |
| 58 | 5F-Cumyl-PEGACLONE | 0,22 | M 30 | Found dead in his apartment; Neither alcohol, nor drugs could be found in the apartment | - | BAC: 0,01  UAC: 0,01 | Most likely cardiotoxic effects of Cumyl-PEGACLONE, considering the deceased’s pre-existing heart condition | Atheromatous plaques in the coronary artery; Significant coronary atheromatosis; Significant pulmonary oedema; Increased brain volume, with little intracranial pressure; Obesity; Blood-filled organs | 1-2 days |  |
| 59 | Cumyl-PEGACLONE | 0,14 | M 44 | Found dead in his apartment, with drug paraphernalia and a box with a white powder; Known intravenous drug abuse habits | Diazepam (77); Nor­dazepam (26); Oxazepam (3,6); Temazepam (5,1); Zolpidem (17); MDPHP (530); *N*-Ethyl­pentylone (2600); 4F-PHP (10) | BAC: 0,01  UAC: 0,02 | Fatal intoxication with *N*-ethyl­pentylone and MDPHP in combination with the other psychoactive substances, like synthetic cannabinoids | Older injection sites on the crook of the left arm; Pronounced pulmonary oedema; Relative cardiac hypertrophy | 2 days |  |
|  | 5F-Cumyl-P7AICA | *Not quant.* |  |  |  |  |  |  |  |  |
| 60 | Cumyl-PEGACLONE | 0,12 | M 46 | Known history of heroin and alcohol abuse; Found dead in his apartment | 6-Monoacetylmorphine (120); Morphine (570); Nor­morphine (*not quantified*); Dihydromorphine (1,4); Codeine (55); Papaverine (6,9); Noscapine (> 50); Amitriptyline (54); Nor­triptyline (22); Pregabalin (4100); Paracetamol (1900) | BAC: 2,17  UAC: 3,09  Elevated levels of methanol in blood and urine | Fatal intoxication with heroin and alcohol. Cumyl-PEGACLONE played a contributory role | Lesion in the groin, which may be an injection site; Moderate intracranial pressure; Pronounced fatty liver cirrhosis | 1-2 days |  |
| 61 | 4F-MDMB-BINACA | *Not quant.* | M 61 | Found dead on the bathroom floor of his apartment; Long history of drug abuse | 6-Monoacetylmorphine (56); Morphine (830); Nor­mor­phine (*not quantified*); Co­de­ine (72); Di­hy­dro­codeine (170); Nos­ca­pine (> 50); Pa­pa­verine (14); Hydro­codone (6,0); Al­pra­zolam (0,8); Diaze­pam (1,8); Nor­da­zepam (8,1); Te­ma­ze­pam (traces); Clo­na­zepam (16); 7-Amino­clo­na­zepam (160); Prega­balin (8300); Paracetamol (5300) | BAC: 0,01  UAC: 0,02 | Aspiration of gastric contents due to an intoxication with heroin and 4F-MDMB-BINACA | Several relatively fresh injection sites on all areas of the body; Moderate haemorrhagic pulmonary oedema; Moderately filled bladder; Pronounced acute intracranial pressure; Airways filled with gastric contents; Cardiac hypertrophy | 19 hours |  |
| 62 | MDMB-4en-PINACA | 0,2 | M 43 | Found dead outside a hospital building with used, disposable syringes, clonazepam and pregabalin; Known history of drug abuse | Clonazepam (7,1); 7-Amino­clonazepam (760); Pregabalin (6200); Mor­phine (traces); Cocaine (traces); Benzoyl­ecgonine (traces); Ecgonine methyl ester (1,4); Paracetamol (traces); Ibuprofen (250); Val-proic acid (1500); Lido-caine (traces); Propofol (230) | BAC: 0,08  UAC: 0,07 | Intoxication with clonazepam, MDMB-4en-PINACA and pregabalin | Rather older injection sites on the left arm; Acute blood-filled inner organs; Increased brain volume with acute intracranial pressure; Pronounced pulmonary oedema, Splenomegaly; Moderate coronary atherosclerosis | 3 days |  |
| 63 | APINACA | 0,88 | M 35 | Found dead in his apartment; History of chronic paranoid schizophrenia and drug and alcohol abuse; Approx. 40 packets of “herbal mixtures”, venlafaxine, olan­zapine and melperone were found at the scene | Venlafaxine (57 000); Norvenlafaxine (4200);  Olanzapine (traces) | BAC: 0,03  UAC: 0,65 | Intoxication with mainly venlafaxine and synthetic cannabinoids | Significant signs of post-mortem decomposition; Several tiny, white tablets in the stomach and small intestine, Approximately 600 mL of urine in the bladder | 2-3 days |  |
|  | JWH-018 | 0,11 |  |  |  |  |  |  |  |  |
| 64 | 5F-AB-PINACA | 0,12 | M 29 | Known history of drug abuse (*e.g.* “bath salts”); Took part in a drug sub­sti­tu-tion programme; Found dead in his apartment with benzodiazepine tablets and an unknown powder in his pocket | Methadone (210); EDDP (21); Tilidine (3,7); Nor­tilidine (1,0); Alprazolam (4,8); 7-Aminoclonazepam (90); Diazepam (52); Nor­dazepam (120); Oxazepam (10); Temazepam (5,5); Lorazepam (55); α-PBP (120) | BAC: 0,01  UAC: 0,01 | Intoxication with α-PBP and the other psychoactive substances detected in blood | Increased intracranial pressure; Pulmonary oedema | 1 day |  |

*Post-mortem interval = Estimated delay between death and sampling of biological specimens

**Table S3.** Summary of cases with a toxicological significance score of 1, for which synthetic cannabinoids could be detected in femoral venous blood.

| Toxicological Significance Score 1 (TSS 1) | | | | | | | | | |
| --- | --- | --- | --- | --- | --- | --- | --- | --- | --- |
| # | Synthetic cannabinoids detected, incl. approximate concentrations in femoral venous blood [µg/L] | | Sex  Age | Case  history | Other substances  detected in  femoral venous  blood  [µg/L] | Blood alcohol concentration (bac) in femoral venous blood  Urine alcohol concentration (uac)  [g/100 mL] | Main cause  of death | Most relevant findings at autopsy | PMI* |
| 65 | 5F-ADB | 0,27 | M 36 | Found dead in the toilet of a bakery with drug para­pher­nalia, incl. a syringe with needle and fentanyl patches; Known history of drug abuse | Fentanyl (42); Norfentanyl (~ 1,0); Morphine (~ 3,1); Hydromorphone (traces); Pregabalin (3700) | BAC: 0,01  UAC: 0,06 | Fatal intoxication with fentanyl and 5F-ADB | Significant haemorrhagic pulmo­nary oedema; Several fresh and older injection sites on all extremities; Pronounced cerebral oedema | 3 days |
| 66 | 5F-ADB | < 0,1 | M 41 | Alcoholic and abuser of drugs; Found dead, lying in his own vomit | Tramadol (720); *N*-Desmethyl­trama­dol (540); O-Desmethyl­tramadol (64); Tilidine (19); Nortilidine (27); Bu­prenorphine (1,0); Nor­bu­pre­nor­phine (0,1); Levetiracetam (2800); Pregabalin (3400); Methyl­phenidate (1,3); Ritalinic acid (190); Pa­ra­cetamol (traces); PV-8 (4,9) | BAC: 0,02  UAC: 0,04  Elevated levels of methanol in blood and urine  EtG in urine: 27 mg/L | Polydrug intoxication, possibly combined with alcohol withdrawal | Two injection sites in the crook of the left arm; Pronounced pulmonary oedema; Fatty liver; Liver cirrhosis | 1 day |
| 67 | 5F-ADB | < 0,1 | M 38 | Found dead in the toilet of a supermarket, with a needle injected in the left hand, and fentanyl patches and utensils for intra­venous drug abuse next to the body | Fentanyl (20);  Norfentanyl (2,0),  Olanzapine (13) | BAC 0,57  UAC 0,81 | Fentanyl intoxication | Advanced state of post-mortem decom­position, including the inner organs; Cardiac hypertrophy (heart weight: 525 g); Signs of pronounced increased intracranial pressure | 4 days |
| 68 | 5F-ADB | < 0,1 | M 28 | Found dead in a friend’s room; History of alcohol and drug abuse | Amphetamine (11) | BAC: 2,52  UAC: 3,12 | Death due to the consumption of amphetamine, 5F-ADB and alcohol in combination with a pre-existing heart condition | Full bladder; Increased brain volume with signs of significant increased in­tra­cranial pressure; Pro­nounced pulmonary oedema; Cardiac hyper­trophy | 7 hours |
| 69 | 5F-ADB | < 0,1 | M 44 | Suddenly fell unconscious after consuming alcohol with friends; The emergency physician attempted to resuscitate, but with no success; “Herbal mixtures” were found at the scene; History of drug (*e.g.* heroin) and alcohol abuse | 6-Monoacetylmorphine (1,2); Morphine (25); Codeine (traces); Citalopram (traces); Mirtazapine (63); Metformin (160); Pipamperone (traces); Paracetamol (190) | BAC: 3,42  UAC: 4,67 | Fatal intoxication with alcohol and heroin | Increased brain volume, with signs of intracranial pressure; Full bladder; Fruity, aromatic odour streaming from the abdominal cavity | 19 hours |
| 70 | 5F-ADB | < 0,1 | M 40 | Extended history of drug abuse; Found dead in his apartment; Was administered lidocaine a few days prior to treat back pain; Metamizole was found in the apartment | 4-Methylaminoantipyrine (1300); 4-Amino­antipyrine (600); Promethazine (13); Lidocaine (*not quantified*) | BAC: 0,05  UAC: 0,04 | Unknown | Signs of acute intracranial pressure; Advanced state of internal and external post-mortem decom­position; Pronounced fatty liver | 4 days |
| 71 | MDMB-CHMICA | < 0,1 | M 50 | Found dead in his bed; Known history of drug abuse | 6-Monoacetylmorphine (10); Morphine (240); Codeine (19); Nordazepam (23); Paracetamol (670) | BAC: 2,82  UAC: 3,82 | Fatal intoxication with heroin and alcohol | Liver cirrhosis; Sig­ni­fi­cant signs of cerebral pressure; Acute increase in brain volume (brain weight: 1428 g); Aroma­tic smell streaming from the abdominal cavity | 1 day |
| 72 | An AB-FUBINACA or AMB-FUBINACA metabolite | *Not quant.* | M 30 | Found dead in his apartment approx. 10 days after he was last seen alive; History of drug abuse; A “bong” was found in the apartment | Quetiapine (700); Flupentixol (66); Trimipramine (2000) | BAC: 0,70  UAC: 1,45 | Fatal intoxication with trimipramine, possibly in combination with quetiapine and flupentixol | Onset of external and internal post-mortem decomposition; In­creased brain volume, with signs of intracranial pressure; Full bladder | 10 days |
|  | 5F-Cumyl-PINACA | < 0,1 |  |  |  |  |  |  |  |
| 73 | 5F-Cumyl-PEGACLONE | 0,17 | F 48 | Found dead in her room, with a used syringe with needle near the body; Morphine, pregabalin, metoclopramide and zo­pi­clone were found in the room; Long history of drug abuse; Underwent drug sub­stitution therapy | Morphine (240); Codeine (traces); Methadone (18); EDDP (3,3); Bupre­norphine (0,27); Norbu­pre­norphine (traces); Pregabalin (810); α-PiHP (16) | BAC: 0,02  UAC: 0,03 | Fatal intoxication with morphine, likely after intravenous administration | Cerebral oedema; Slimy material in the airways; Significant signs of aspi­ration of stomach con­tents in both lung lobes; Little gastric contents; ~ 300 mL of urine in the bladder | 2 days |
| 74 | EG-018 | < 0,1 | M 33 | History of drug abuse; Found dead in the early hours of the following morning after consuming buprenor­phine, synthetic cannabi­noids and alcohol; Marihuana and used syringes were found in the apartment | 6-Monoacetylmorphine (12); Morphine (180); Nor­morphine (*not quantified*); Codeine (18); Papaverine (3,7); Nos­capine (> 50); Mirtazapine (traces); Paracetamol (360) | BAC: 0,00  UAC: 0,01 | Fatal intoxication with heroin | Moderate inhalation of gastric contents into the upper airways; Acute hyperaemia of the liver, spleen and kidneys; Signs of intracranial pressure; 150 mL of urine in the bladder | 3-4 days |
|  | Cumyl-PEGACLONE | 0,94 |  |  |  |  |  |  |  |
| 75 | EG-018 | *Not quant.* | F 48 | Complaints were issued about an unpleasant smell coming from the deceased’s apartment, after which the apart­ment was accessed by the police; The deceased was found dead, in an advanced state of de­com­position; Known history of drug abuse; Drug paraphernalia were found in the apartment | 6-Monoacetylmorphine (3,7); Morphine (500); Normorphine (*not quan­tified*); Codeine (36); Pa­pa­verine (5,9); Nosca­pine (5,9); Methadone (29); EDDP (traces); Tilidine (5,8); Nortilidine (traces); Diazepam (1350); Nor­da­ze­pam (560); Oxa­zepam (traces); Temazepam (60); Pregabalin (4500); Cita­lo­pram (95); Mirta­za­pine (330); Doxepin (8,1); Nor­dox­e­pin (49); Pi­pam­perone (12); Pro­thi­pendyl (*not quantified*); Chlor­pro­thi­xene (4,4); Para­cet­a­mol (1200) | BAC: 2,10  Elevated levels of methanol in blood | Intoxication with heroin, in combination with the other central nervous system depressing drugs | Significant signs of post-mortem decomposition with maggot infestation; Advanced liver cirrhosis; Gall stones | 18 days |
| 76 | Cumyl-PEGACLONE | 0,4 | M 37 | Found dead in his room in a homeless centre; Known history of drug abuse and coronary heart disease; Several pharmaceuticals, “herbal mixtures” and a syringe were found in the room | Alprazolam (100); Hy­dro­xy­alprazolam (4,5); Dia­ze­pam (traces); Nor­da­ze­pam (traces); Metha­done (380); EDDP (32); Fen­tanyl (traces); Mirta­za­pine (65); Quetiapine (190); Pregabalin (1600); Ra­mi­prilat (1,6); Bi­so­pro­lol (5,5); Ambroxol (33) | BAC: 0,01  UAC: 0,02 | Aspiration of stomach contents, pre-existing heart condition and an intoxication with the detected central nervous system depressing substances, incl. Cumyl-PEGACLONE | Autolytic changes to the spleen and liver; Mo­de­rate­ly fatty liver; Post-mortem decomposition of the lungs; Bloody pul­mo­na­ry oedema; Coronary artery stenosis; Older signs of cardiac infarction | 4 days |
| 77 | Cumyl-PEGACLONE | 0,38 | F 21 | Found in a comatose state, lying next to her boyfriend; Resuscitation efforts by emergency physician failed; Known history of drug abuse and methadone substitution therapy; Was prescribed bromazepam, clona­ze­pam and pregabalin by her physician | 6-Monoacetylmorphine (18); Morphine (200); Nor­morphine (*not quantified*); Co­de­ine (9,2); Noscapine (*not quantified*); Papaverine (*not quan­ti­fied*); Prega­ba­lin (14 000); Bro­ma­ze­pam (190); Hy­droxy­bro­ma­zepam (*not quant­ified*); 7-Amino­clo­na­ze­pam (170); Di­a­ze­pam (88); Nor­da­ze­pam (72); Oxazepam (9,4); Te­ma­ze­pam (11); Pa­racetamol (110); Meth­amphetamine (45); Am­phetamine (17) | BAC: 0,01  UAC: 0,02 | Fatal intoxication  with heroin | Fresh injection sites on the crook of the right arm; Older injection sites on the back of the left hand; Significant increase in brain volume, with signs of intracranial pressure; Acute passive hyperaemia of the inner organs | 12 hours |
| 78 | AMB-CHMICA | < 0,1 | M 39 | Found dead in his apartment; Known history of drug abuse and substitution therapy with methadone; The deceased used to crush and apply the metha­done tablets intra­ve­nously | Methadone (780); EDDP (100); Fentanyl (0,7); Norfentanyl (traces); Mirtazapine (24); Pregabalin (600); Flupirtine (200); Lidocaine (*not quantified*); U-47700 (indication) | BAC: 0,01  UAC: 0,01 | Fatal intoxication with mainly methadone | Several injection sites in the left groin; Acute hyperaemia of the inner organs; Increased brain volume with signs of acute intracranial pressure; Cardiac hypertrophy (heart weight: 519 g) | 1 day |
| 79 | JWH-122 | < 0,1 | M 19 | Known history of drug abuse, incl. “herbal mixtures”; Went missing for 11 days after leaving home; Found dead in an abandoned house, where he hung himself | - | BAC: 0,13  UAC: 0,55  Significantly elevated levels of methanol in urine | Suicide by hanging | Early stages of post-mortem decomposition; Ligature furrow visible externally and internally; No signs of violence to the body by a third party | 40 days |
| 80 | 5F-APINACA | < 0,1 | M 24 | History of narcotics abuse; Found in a comatose state in his room, with an E-ciga­ret­te, a white powder and drug paraphernalia; Transported to the local hospital; Un­success­ful efforts to reanimate | Lidocaine (*not quantified*); MT-45 (2900); Methox­met­amine (indic­at­i­on) | BAC: 0,00  UAC: 0,00 | Fatal intoxication  with MT-45 | Clear signs of intracranial pressure; Acute haemorrhagic organs; Filled bladder; Little gastric contents | 20 hours |
|  | PB-22 | < 0,1 |  |  |  |  |  |  |  |
| 81 | 5F-MDMB-P7AICA | < 0,1 | M 45 | Presumably HIV positive; Found lying underneath a bridge; Emergency physician was unable to resuscitate; A syringe with brownish fluid was found next to the body | 6-Monoacetylmorphine (5,7); Morphine (100); Co­deine (8,0); Nos­ca­pine (*not quantified*); Pa­pa­verine (*not quantified*); Oxa­zepam (500); Zopi­clone (traces); Pre­ga­ba­lin (490); Levetiracetam (450); Trimipramine (7,5); Mirtazapine (12); Bi­soprolol (traces);  Pa­racetamol (370) | BAC: 0,14  UAC: 0,15 | Aspiration of gastric contents following an intoxication with heroin and the other central nervous system depressing agents | Negative test for HIV; Signs of increased intracranial pressure; Pronounced fatty liver; Full bladder; Injection sites in the crook of the left arm; Two fresh injection sites in the crook of the right arm | 16 hours |

*Post-mortem interval = stimated delay between death and sampling of biological specimens

**Table S4.** Summary of cases, for which synthetic cannabinoids were detected in specimens other than femoral venous blood.

| # | Alternative specimen | Synthetic cannabinoids, incl. concentrations detected in liver [µg/kg] or blood [µg/L] | | | Sex  Age | Case history | Other substances detected in liver [µg/kg] or blood [µg/L] | Alcohol concentration in the indicated specimen  [g/100 mL or g/100 g] | Main  cause  of death | Most relevant findings at autopsy | *Time until sampling |
| --- | --- | --- | --- | --- | --- | --- | --- | --- | --- | --- | --- |
| Toxicological Significance Score 3 (TSS 3) | | | | | | | | | | | |
| 82 | Liver | 5F-ADB | 7,2 | | M 53 | Body was found in an advanced state of decomposition and infested with larvae in the deceased’s apart­ment; The deceased was last seen 6 days prior to discovery | Liver: Methadone (11 000); EDDP (2100); Mor­phine (100); Nor­mor­phine; Codeine; Nor­co­de­ine; Noscapine; Papa­ve­rine (*not quant.*); Pre­ga­balin (45 000); Nor­da­ze­pam (66); Oxa­ze­pam (15 000); Lora­zepam (1300); Mel­perone (17); Pa­ra­cet­a­mol (230) | Muscle: 0,86 | Intoxication with methadone and synthetic cannabinoids | Significantly advanced state of decomposition, with maggot infestation; Moderate general atheromatosis; Cholestasis of the gall bladder | 7 days |
|  |  | AMB-FUBINACA | 100 | |  |  |  |  |  |  |  |
| 83 | Cardiac  blood | 5F-ADB | 0,16 | | M 25 | Did not return home after taking the dog for a walk; His body was found in a river nearly 2 months later; Known consump­tion of “herbal mixtures” | - | Muscle: 0,32 | Drowning, likely resulting from central nervous system depression after 5F-ADB consumption | Significant external and internal post-mortem decomposition; Signs that the body was sub­merged in water for a long time, Abnormal distention of both lungs | 47 days |
| 84 | Cardiac  blood | 5F-MDMB-PICA | | 1,70 | M 47 | Known alcohol and drug abuse (*e.g.* marihuana, amphetamine and heroin); Found dead in his apartment, with his head hanging deep inside the toilet | Femoral venous blood:  Pregabalin (4600); 6-Mo­no­acetylmorphine (tra­ces); Codeine (traces); Pa­racetamol (35); Am­phet­amine (14) | Femoral venous blood:  BAC: 1,66  Significantly elevated levels of methanol in blood | Direct or indirect (aspiration of gastric contents) result of an intoxication with 5F-MDMB-PICA, possibly with heroin, pregabalin and alcohol | Significant post-mortem de­com­position and auto­ly­sis; Post-mortem lividi­ty, especially on the front side of the upper body; Significant amounts of gastric contents in the deeper lung sections; Significant cyanosis of the inner organs; Fatty liver | 3 days |
|  |  | A 4F-MDMB-BINACA hydrolysis product | | *Not quant.* |  |  |  |  |  |  |  |
| 85 | Cardiac  blood | 5F-MDMB-PICA | 0,43 | | F 40 | Administered to hospital after collapsing at home; Declared dead approx. 1 hour later; History of consuming drugs (*e.g.* “herbal mixtures”) | Femoral venous blood:  Doxylamine (8,1); Lido­caine (*not quan­ti­fied*); Atropine (13); Amio­da­rone (59); *N*-Des­ethyl­amiodarone (71) | BAC: 0,02  UAC: 0,09 | Possibly cardiotoxic effects of synthetic cannabinoids, conside­ring the deceased’s pre-existing heart condition | Pulmonary interstitial oede­ma; Cerebral oedema; Cardiac hyper­trophy; Significant coronary arterio­sclerosis | 1-2 days |
|  |  | A 4F-MDMB-BINACA hydrolysis product | *Not quant.* | |  |  |  |  |  |  |  |
|  |  | MDMB-4en-PINACA | < 0,1 | |  |  |  |  |  |  |  |
| 86 | Cardiac blood | MDMB-4en-PINACA | 0,8 | | M 47 | Long history of drug and alcohol abuse; Found dead in bed by his girlfriend approximately 2 hours after falling asleep | Femoral venous blood:  Buprenorphine (4,0); Nor­buprenorphine (2,3); Al­pra­zolam (50); Hy­droxy­alprazolam (2,9); Di­azepam (15); Nor­da­ze­pam (40); Te­ma­ze­pam (1,2); Pregabalin (7300) | Femoral venous blood:  BAC: 2,17  UAC: 2,99  Significantly elevated levels of methanol in blood and urine | Intoxication with  MDMB-4en-PINACA  and alcohol | Small heart, with little coronary atherosclerosis; Slightly distended atria; Significant pulmonary em­phy­sema; Splenomegaly | 9 hours |
|  |  | 4F-MDMB-BICA hydrolysis product | *Not quant.* | |  |  |  |  |  |  |  |
| 87 | Antemortem serum taken in hospital | 5F-MDMB-PICA | 0,20 | | F 34 | Known history of drug abuse; Underwent methadone substitution therapy; Collapsed at the railway station after con­su­ming methadone, ben­zo­diazepines and “Spice”; Hospital ad­mis­sion, Declared brain dead a week later | Methadone (26); EDDP (1,7); Lorazepam (60); Que­tiapine (traces); Pre­ga­balin (110); 9-Hy­droxy­risperidone (tra­ces); Metoprolol (traces); U­rapidil (660); Sufentanil (0,19) | BAC: < 0,2 | Intoxication with  5F-MDMB-PICA, lorazepam, pregabalin and methadone | Heart and kidneys removed for transplant purposes; Significant brain volume increase; Signs of intensive care measures | 33 hours |
| 88 | Antemortem whole blood taken in hospital | ADB-CHMINACA | 3,9 | | M 27 | Found unconscious, lying in his own vomit; Transported to the hospital, where he died 6 days later | THC-COOH (16); Lidocaine (*not quantified*) | BAC: 1,13  UAC (post-mortem urine): 1,99  Elevated levels of methanol in blood and urine | Aspiration of stomach contents after intoxi­ca­ti­on with ADB-CHMINACA and alcohol | Signs of aspiration, Bronchitis; Disintegrating brain; Signs of intensive care measures | 1,5 hours |
| 89 | Cardiac blood | 5F-Cumyl-PEGACLONE | 1,5 | | M 36 | The decomposed body was found in the de­ceased’s bed; Known his­tory of drug abuse and schizophrenia; Drug paraphernalia and “herbal mixtures” were found in the apartment | Femoral venous blood:  Morphine (78); Hydro­mor­phone (traces); Mel­pe­rone (54); Pregabalin (940); Risperidone (traces); 9-Hydroxy­ris­pe­ri­done (traces); Ven­la­fax­ine (260); *N*-Des­methyl­venlafaxine (67) | Femoral venous blood:  BAC: 0,17  UAC: 0,44 | Intoxication with  5F-Cumyl-PEGACLONE and morphine, in combination with the other central nervous system depressants detected in blood | Advanced signs of post-mortem decomposition; No signs of violence to the body | 5 days |
| 90 | Cardiac blood | EG-018 | 14 | | M 36 | Found dead in his bed; The state the apartment was found in suggested that a physical struggle had taken place; Known history of drug abuse | - | Femoral venous blood:  BAC: 0,47  Significantly elevated levels of methanol, acetone and 2-propanol in blood | Most probably an intoxication with EG-018, in combination with alcohol withdrawal | Advanced state of internal and external post-mortem decompo­si­ti­on; Fatty liver; Pneumothorax both sides | 2-6 days |
| Toxicological Significance Score 2 (TSS 2) | | | | | | | | | | | |
| 91 | Liver | Cumyl-4CN-BINACA | 1,8 | | M 34 | Body was found in a sig­ni­ficant state of decom­po­sition in his apartment approx. 1 month after the deceased was last seen alive; Known histo­ry of drug abuse; Drug paraphernalia were found at the scene | Fentanyl (110); Tilidine (100); Nortilidine (550); Naloxone (22); Diphen­hy­dramine (39) | Muscle: ~ 1,2 | Fatal intoxication with fentanyl in combination with the other central nervous system depressing drugs detected in the liver | Significant post-mortem decomposition with maggot infestation | 31 days |
| 92 | Cardiac blood | Cumyl-CBMICA | 4,5 | | M 43 | Known history of drug abuse; Died approx. 10 minutes after hospital admission; Consumed alcohol with his friend beforehand; Fentanyl patches were found in the apartment | Femoral venous blood:  Fentanyl (11); Nor­fen­ta­nyl (*not quantified*); Pa­ra­cetamol (34); Atropine (3,2) | Femoral venous blood:  BAC: ~ 1,3  UAC: 1,70 | Fatal intoxication with fentanyl, possibly in combination with Cumyl-CBMICA and alcohol | Gastric contents in the laryngeal inlet and bran­ches of the airways; In­creased brain volume, with signs of supra­ten­to­ri­al intracranial pressure; Blood-filled lungs | 2-3 days |
| 93 | Cardiac blood | MDMB-CHMICA | 0,10 | | M 36 | Found dead in his apartment, with a syringe close to the body; Drug parapherna­li­a, incl. syringes, a spoon and the packaging of a fentanyl patch were found at the scene; Long history of drug abuse known | Femoral venous blood:  Fentanyl (85); Nor­fentanyl (10); Lorazepam (550); 7-Amino­clona­ze­pam (in a low concentration); Prega­ba­lin (3200) | Muscle: 0,82 | Intoxication with fentanyl | Advanced state of external and internal post-mortem decompo­si­ti­on; 150 mL of stomach contents; No urine | 3 days |
| 94 | Cardiac blood | 5F-Cumyl-PEGACLONE | 0,31 | | M 26 | The deceased rode into a brick wall on a scooter with full speed while not wearing a helmet; Declared dead on the scene | Femoral venous blood:  Amphetamine (80); Meth­amphetamine (tra­ces); Cocaine (3,6); Ec­go­nine methyl ester (10); Ben­zo­yl­ecgonine (21); Co­ca­ethy­lene (traces) | Femoral venous blood:  BAC: 0,39  UAC: 0,57 | Traumatic brain injury and severe blood loss after a traffic accident, likely resulting from a significant intoxication | Pronounced traumatic brain injury; Destruction of the neurocranium; Epidural and subdural bleeding; Anaemia of the inner organs, Little blood in the upper airways | 1 day |
| Toxicological Significance Score 1 | | | | | | | | | | | |
| 95 | Cardiac blood | 5F-ADB | < 0,1 | | M 22 | The deceased was a known heroin and alcohol addict; Found dead in his apartment | Femoral venous blood:  6-Monoacetylmorphine (2,7); Morphine (82); Hy­dro­morphone (0,4); Co­de­ine (22), Bu­pre­nor­phine (0,31); Alprazolam (0,9); 7-Amino­clo­na­ze­pam (5,1); Diazepam (170); Nordazepam (120); Oxazepam (7,3); Te­mazepam (8,9); Pre­ga­balin (1200); Pa­ra­cet­a­mol (500); Bupropion (7,6); Ritalinic acid (4,3) | Femoral venous blood:  BAC: 0,01  UAC (0,04) | Fatal intoxication with heroin, possibly in combination with the other central nervous system depressants detected in blood | 1,5 L of urine in the bladder; Significant pulmonary oedema, especially of the right lobe; Increased brain volume, with signs of acute intracranial pressure | 2 days |
| 96 | Cardiac blood | 5F-MDMB-PICA | < 0,1 | | M 38 | Found dead in his apart­ment, with a used syringe lying near the body, Marihuana was found in the apartment; Known history of drug abuse | Femoral venous blood:  6-Monoacetylmorphine (14); Morphine (260); Co­deine (14); Meth­a­done (9,8); EDDP (1,2); Pre­gabalin (7900); Que­ti­apine (370); Am­pheta­mine (100) | Femoral venous blood: BAC 0,14  UAC: 0,22 | Intoxication with heroin | Onset of internal post-mortem decomposition; Fresh injection sites on the back of the left hand; Increased brain volume, with signs of moderate intracranial pressure; Pulmonary oedema | 1 day |
|  |  | MDMB-4en-PINACA | < 0,1 | |  |  |  |  |  |  |  |
| 97 | Urine taken in hospital | An AB-FUBINACA or AMB-FUBINACA metabolite | *Low conc.* | | M 31 | Declared brain dead three days after ad­missi­on to the ICU; No blood from the day of admission - only urine - was available for toxicological analysis | Urine: 6-Mono­acetyl­mor­phine, Morphine, Nor­mor­phine, Codeine, Nor­co­deine, Dihydro­mor­phine, Noscapine, Pa­pa­ve­rine, Para­cet­a­mol, Su­fen­tanil, Mi­da­zo­lam, Hy­d­roxy­midazolam, Le­ve­ti­ra­cetam, Fu­ro­se­mide, Am­picillin, Pipera­cil­lin, Li­docaine, Pro­po­fol, Ni­co­tine, Cotinine, Caf­fe­ine, 4-Methyl-α-PHP (*not quantified*) | UAC 0,01 | Intoxication with heroin, possibly combined with 4-Methyl-α-PHP | Increased brain volume, with signs of acute su­pra­tentorial pressure; Pronounced pulmonary oedema; Little general atheromatosis | 2 hours |
| 98 | Cardiac blood | A 4F-MDMB-BINACA hydrolysis product | *Not quant.* | | M 32 | Long history of narcotics abuse; The deceased took part in a drug substitution programme; Ex­pe­ri­en­ced trouble sleeping; Found dead in his apartment | Femoral venous blood:  Methadone (750); EDDP (100); Phenobarbital (350); Alprazolam (1,1); Lo­razepam (190); Di­a­ze­pam (traces); Nor­da­ze­pam (14); Oxazepam (390); Clo­nazepam (traces); 7-Amino­clo­na­ze­pam (170); Pregabalin (6500); 6-Mono­ace­tyl­mor­phine (traces); Mor­phine (0,8) | BAC: 0,00  UAC: 0,02 | Intoxication with methadone and the other central nervous system depressing agents detected in femoral venous blood | Cyanosis of the inner organs and mucous membranes; 500 mL of urine in the bladder; Significant cyanosis of the brain; No signs of fresh injection sites in the groin area; Pulmonary oedema | 1 day |

*Estimated delay between death and sampling of post-mortem specimens, or between the intoxication incident and sampling of blood or urine in hospital
